# Dairy Consumption and Risk of Metabolic Syndrome: Results from Korean Population and Meta-Analysis

**DOI:** 10.3390/nu13051574

**Published:** 2021-05-08

**Authors:** Shaoyue Jin, Youjin Je

**Affiliations:** Department of Food and Nutrition, Kyung Hee University, Seoul 02447, Korea; shaoyue666@gmail.com

**Keywords:** metabolic syndrome, dairy products, milk, yogurt, cheese

## Abstract

Dairy consumption has been associated with decreased risk of metabolic syndrome (MetS) in previous studies, but the association may be different according to each type of dairy products and its subgroups. Thus, we conducted an updated meta-analysis of observational studies to examine the association between various dairy products and risk of MetS. The PubMed and Web of Science databases were searched for eligible studies published up to February 2021. In addition, we included unpublished results from Korea National Health and Nutrition Examination Survey, 2013–2018, including 23,319 Korean adults and the elderly. A total of 35 studies (12 cohort studies and 25 cross-sectional studies) with 398,877 subjects were included in the meta-analysis. The pooled relative risks (RR) of MetS for the highest versus lowest categories of dairy consumption was 0.80 [95% confidence interval (CI): 0.72–0.88]. For the type of dairy products, there were also significant inverse associations with milk (RR: 0.83; 95% CI: 0.78–0.89) and yogurt consumption (RR: 0.89; 95% CI: 0.83–0.95). For cheese consumption, however, no significant association was found (RR: 0.98; 95% CI: 0.86–1.11). Our findings suggest that milk and yogurt consumption is inversely associated with the risk of MetS, but not cheese consumption.

## 1. Introduction

Metabolic syndrome (MetS) is a complex of interrelated risk factors for cardiovascular disease and diabetes, including raised blood pressure, raised triglycerides, lowered high-density lipoprotein cholesterol, raised fasting glucose, and central obesity [1]. About 20–25% of the world’s adult population has MetS [2]. Compared to people without MetS, those with MetS are twice as likely to increase the risk of cardiovascular disease (CVD) and CVD mortality, and 1.5 times as likely to increase the risk for all-cause mortality [3]. The relationship between dairy consumption and the risk of MetS has already been extensively investigated, and there are some hypotheses that some ingredients in dairy products, including a variety of minerals, dairy proteins, and dairy fats, may have beneficial effects on MetS [4,5].

Four meta-analyses on dairy consumption and MetS risk have been previously conducted [6,7,8,9]. Two meta-analyses were mainly conducted on total dairy consumption [6,7], while the other two meta-analyses reported some results of individual dairy products but did not conduct subgroup analyses of individual dairy products [8,9]. Previous studies did not perform stratified analyses by sex and criteria for MetS, and previous meta-analyses pointed out the different MetS criteria as a limitation [7,8,9]. In addition, there were some observational studies on cheese consumption and risk of MetS, but no meta-analysis of cheese consumption on risk of MetS has been conducted to date. Also, we need to conduct an updated meta-analysis of observational studies with the most recent data and dose-response analyses.

Therefore, we systematically reviewed and performed a comprehensive meta- analysis to quantitatively evaluate the association between total dairy products and subtype of dairy products, such as milk, yogurt and cheese consumption, and MetS. In addition, we analyzed the associations of each type of dairy products with the risk of MetS using the data of Korea National Health and Nutrition Examination Survey (KNHANES) and included these new results in our meta-analysis.

## 2. Materials and Methods

### 2.1. Korea National Health and Nutrition Examination Survey (KNHANES) Data

#### 2.1.1. Study Participants

The present study was based on the KNHANES data, which was conducted by the Korea Centers for Disease Control and Prevention (KCDC) under the Korean Ministry of Health and Welfare. A total of 47,217 participants were collected from the KNHANES 2013–2018, and among them, 39,642 subjects who completed the health interview, health examination, and nutrition survey were included. The following individuals were subsequently excluded: 8453 participants who were <19 years old; 2916 participants who had previously diagnosed with a stroke, myocardial infarction, or cancer; 409 participants who were pregnant or lactating women; 536 participants who had extreme energy intake (<500 or >5000 kcal/day); 1331 participants who had no fasting status at blood test; 1164 participants who had incomplete information on biomarkers; 1514 participants who had missing data on alcohol consumption, smoking, or moderate or vigorous physical activity. Finally, 23,319 participants (18,206 adults, 5113 elderly people) were included in the analysis of MetS. The Institutional Review Board of the KCDC provided formal ethics approval for the KNHANES dataset (2013-07CON-03-4C, 2013-12EXP-03-5C, 2018-01-03-P-A), and informed consent was obtained for each participant in the survey.

#### 2.1.2. Dietary Assessment

Milk and yogurt consumption was assessed from a 24 h dietary recall based on the coding scheme of KNHANES [10,11]. According to the 2015 Dietary Reference Intake for Koreans (KDRI), one serving was defined as 200 g for milk, 150 g for liquid yogurt, 100 g for semisolid yogurt [12]. The consumption of milk and yogurt was categorized into three groups (0, <1, ≥1 serving/day).

#### 2.1.3. Assessment of Metabolic Syndrome (MetS)

The MetS was defined using the National Cholesterol Education Program Adult Treatment Panel III (NCEP ATP III) [13] and the Korean Society for the study of Obesity [14] criteria. The MetS was diagnosed when at least three of the following five components were present: (1) abdominal obesity (waist circumference ≥90 cm for men and ≥85 cm for women); (2) elevated blood pressure (systolic blood pressure ≥130 mmHg or diastolic blood pressure ≥85 mmHg or use of antihypertensive medication); (3) low high-density lipoprotein (HDL)-cholesterol (fasting HDL-cholesterol <40 mg/dL for men and <50 mg/dL for women); (4) hypertriglyceridemia (fasting triglyceride ≥150 mg/dL); (5) hyperglycemia (fasting plasma glucose ≥100 mg/dL or physician’s diagnosis or use of insulin or oral antidiabetic medication).

#### 2.1.4. Confounding Variables

Information on the demographic and lifestyle factors including age, sex, socioeconomic, smoking status, alcohol consumption, and physical activity were obtained by personal interview or using a self-administered questionnaire. Education level was classified as “≤middle school”, “high school”, and “≥college”. Household income level was categorized into the lowest, lower-middle, upper-middle, and highest. Smoking status was divided into the non-smoker, former smoker, and current smoker. We surveyed drinking experience in the past year, and alcohol consumption was categorized into never/rarely, 1–4 times/month, and ≥2 times/week. Physical activity was assessed by the practice of aerobic activity. The practice of aerobic activity was defined as ≥150 min/week of moderate physical activity, ≥75 min/week of vigorous physical activity, or ≥150 min/week of a combination of moderate and vigorous physical activity (1 min of vigorous physical activity was considered 2 min of moderate physical activity). Furthermore, we also calculated total energy intake and used it as a continuous variable.

#### 2.1.5. Statistical Analysis

The KNHANES data were combined from 2013–2018 while considering the cluster sampling design for the survey according to the guidelines provided by KCDC [10,11]. The general characteristics of the participants were described as shown in Supplemental Tables S1–S3. Continuous variables such as age, body mass index (BMI), total energy intake, nutrient intake, and milk and yogurt intake were expressed as means ± standard errors (SEs), and categorical variables such as sex, education level, household income, smoking status, alcohol consumption and physical activity were expressed as numbers of participants and percentages. The differences in characteristics according to milk and yogurt consumption were evaluated and the PROC SURVEYREG procedure for continuous variables and the chi-square test for categorical variables were used. In addition, the PROC SURVEYLOGISTIC procedure was used for logistic regression analysis and age-adjusted and multivariable-adjusted odds ratios (ORs) and 95% confidence intervals (CIs) of MetS were calculated according to the milk and yogurt consumption. All statistical analyses were performed using SAS software, version 9.4 (SAS Institute Inc., Cary, NC, USA). A two-tailed *p* value < 0.05 was considered to indicate statistical significance.

### 2.2. Systematic Review, Meta-Analysis

#### 2.2.1. Literature Search and Study Selection

Eligible studies were searched from published up to February 2021 in PubMed and ISI Web of Science databases. The following search terms were used: “(milk, yogurt, yoghurt, cheese, or dairy) combined with (metabolic syndrome, syndrome X, or insulin resistant syndrome)”. In addition, a manual search of the reference lists of all relevant studies was conducted, and the articles were reviewed to find other qualified studies. This meta-analysis included prospective cohort studies and cross-sectional studies to report the association between consumption of dairy products and risk of MetS, and included articles published in full-length and in English. To be included in the analysis, the study reported relative risk (RR) and 95% confidence intervals (CI). If there were several publications from the same study, we selected the publication with the largest number of cases or selected a cohort study instead of a cross-sectional study.

#### 2.2.2. Data Extraction

Two investigators (S.J. and Y.J.) extracted data independently, according to the meta-analysis of observational studies in epidemiology (MOOSE) guidelines [15], and any disagreements were resolved by further discussion and referencing the original articles. The following data were extracted from each study: first author’s last name, year of publication, geographic region, study design, follow-up period or study period, baseline age, sex, sample size, number of cases, type of dairy products, adjustment factors, and RRs and 95% CIs for the association between consumption of dairy products and MetS across various categories of exposure or per unit change in exposure. If the study provided more than one RR from age-adjusted models to different multivariate models, we used the RR of the multivariate model to make the most full adjustment for confounders.

#### 2.2.3. Statistical Analysis

The pooled RRs and 95% CIs of the highest versus lowest dairy products consumptions were calculated from the original studies. The natural logarithm of the RRs from the original study were combined through using the DerSimonian and Laird random-effects models, which incorporate both within- and between-study variations [16]. If the original study did not report the lowest level as a reference, we recalculated the RR and its 95% CI [17,18,19,20,21]. When a study reports the results of normal weight at base line and overweight or obese at baseline, we first combined the two results using a fixed-effect model to obtain an overall estimate before combining with other studies [22,23]. If a study reported individual RR for whole milk and low-fat milk but not total milk, we included the effect estimates of whole milk in the main analysis of milk [24,25]. We presented the summary estimates as forest plots. Statistical heterogeneity among the studies was assessed using the *Q* statistic [26] and inconsistency was quantified through the *I*^2^ statistic [27]. We conducted subgroup analyses stratified by study design (cohort/cross-sectional), sex, geographic region (Europe/United States/Asia/Oceania), and criteria for MetS (NCEP ATP III/Joint Interim Statement (JIS)/International Diabetes Federation (IDF)/others).

For the dose-response analysis, a generalized least-squares trend (GLST) estimation, based on the method developed by Greenland and Longnecker, was used to compute study-specific slopes from the natural logs of the RRs across intake categories of dairy product consumption [28,29,30]. The method requires the number of cases and subjects or person-years and RRs with variance estimates for at least three levels of dairy products consumption. For this analysis, prospective cohort studies were included only. The servings, portions, or ounces of dairy product intake per day, week, or month were converted to grams per day for these analyses. According to the standard of ‘United States Department of Agriculture Food and Nutrient Database for Dietary Studies’ [31], we defined one serving as 177 g for dairy products, 244 g for milk, 244 g for yogurt, and 43 g for cheese. Finally, potential publication bias was assessed with Begg’s [32] and Egger’s tests [33]. A two-sided *p* value < 0.05 was considered statistically significant. Stata/SE version 14.2 Software (StataCorp, College Station, TX, USA) was used for all statistical analyses.

## 3. Results

### 3.1. KNHANES Analysis

The general characteristics of the study population according to milk and yogurt consumption are shown in Supplementary Tables S1–S3. More than three-quarters of the adult and elderly population did not consume milk or yogurt. The people who consumed higher levels of milk or yogurt had higher calcium intake than non-consumers. For adult and elderly people, frequent milk and yogurt consumers had a higher proportion of total energy intake from fat but had a lower proportion of total energy intake from carbohydrates, while these correlations were stronger in elderly people.

The associations of milk consumption with risk of MetS in adults and elderly people are presented in Table 1. After adjusting for covariates, high milk consumption was associated with 26% lower odds of MetS in elderly people compared to no milk consumption (OR: 0.74; 95% CI: 0.57–0.96; *p* for trend = 0.029), but no association was found in adults (OR: 0.91; 95% CI: 0.78–1.06; *p* for trend = 0.246). For the elderly, when stratified by sex, a similar inverse association was found in women (OR: 0.72; 95% CI: 0.52–0.99; *p* for trend = 0.058), but not in men (OR: 0.83; 95% CI: 0.56–1.23; *p* for trend = 0.359). For yogurt consumption, however, there was no significant association with risk of MetS (Table 2).

The associations of milk and yogurt consumption with MetS components are shown in Supplementary Tables S4 and S5. For milk consumption, high milk consumption was inversely associated with hypertriglyceridemia in adult men (OR: 0.77; 95% CI: 0.66–0.90; *p* for trend = 0.002) and low HDL-cholesterol in elderly women (OR: 0.63; 95% CI: 0.48–0.84; *p* for trend = 0.002). For yogurt consumption, high yogurt consumption was inversely associated with hyperglycemia in adult women (OR: 0.76; 95% CI: 0.61–0.94; *p* for trend = 0.006) and elderly men (OR: 0.65; 95% CI: 0.43–0.98; *p* for trend = 0.049).

### 3.2. Systematic Review and Meta-Analysis

#### 3.2.1. Study Characteristics

**Figure 1:**
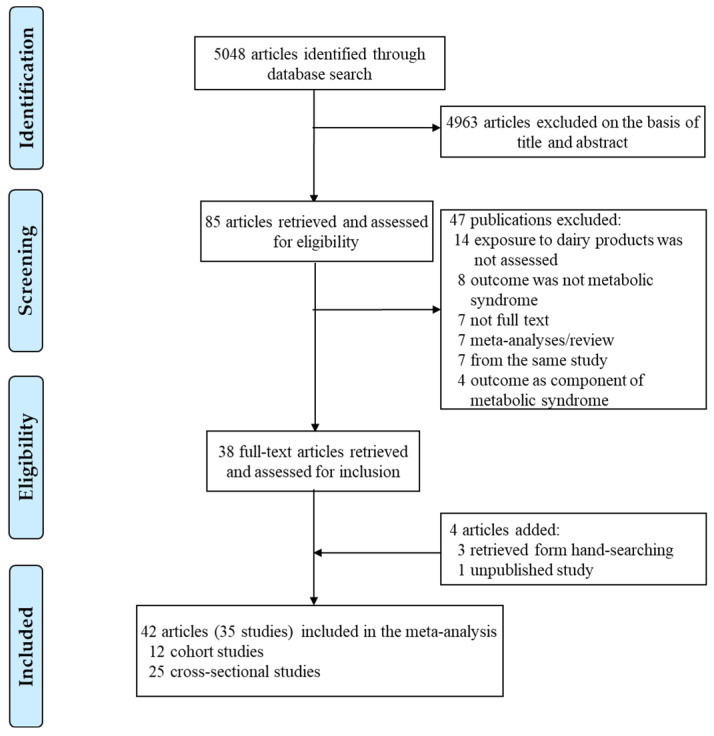
Flow chart of the study selection.

A total of 35 studies including 12 cohort studies [22,23,24,34,35,36,37,38,39,40,41,42,43,44] with 40,943 subjects and 9436 cases and 25 cross-sectional [17,18,19,20,21,25,45,46,47,48,49,50,51,52,53,54,55,56,57,58,59,60,61,62,63,64,65] studies with 362,196 subjects were included in meta-analyses of the highest versus the lowest dairy product consumption and risk of MetS (Figure 1). Table 3 presents the characteristics of studies included in the meta-analysis. By geographic region, 16 studies were performed in Asia, 9 studies in America, 8 studies in Europe, 1 study in Oceania, and 1 study was a multinational study. The participants were all aged ≥18 years at baseline, and durations of follow-up of cohort studies ranged from 2.05 to 20 years, and the mean follow-up period was 7.22 years. The defined MetS in the studies was mostly from NCEP ATP III, JIS, or IDF criteria. Twenty-one studies used NCEP ATP III or modified NCEP ATP III criteria, 12 studies used JIS criteria, and 4 studies used IDF criteria. The two studies reported the results using several different MetS criteria [38,54]. All of studies adjusted for age, and most of the studies adjusted for BMI (kg/m^2^), smoking, alcohol intake, physical activity, socioeconomic status, and total energy intake.

#### 3.2.2. Total Dairy Consumption and MetS

Twenty-two studies investigated the association between the highest versus the lowest total dairy consumption and risk of MetS, and included 210,790 participants (Table 3). The pooled RR for all studies was 0.80 (95% CI: 0.72–0.88) (Table 4), with some evidence of substantial heterogeneity (*I*^2^ = 82.0%, *p <* 0.001). A similar inverse association was found in cohort and cross-sectional studies (Table 4, Figure S1a). By sex, the inverse association tended to be stronger in women than men. By geographic region, there was a significant inverse association in America, Asia, and Europe, while only one study from Oceania showed no significant association. Regarding the criteria of MetS, a significant inverse association was shown in NCEP ATP III, JIS, and other criteria but not in IDF criteria. Furthermore, the heterogeneity disappeared in JIS (*I*^2^ = 20.2%, *p* = 0.28), IDF (*I*^2^ = 57.3%, *p =* 0.07), and other criteria (*I*^2^ = 31.3%, *p* = 0.22) when stratified by the criteria. When we conducted a meta-regression analysis to assess the variations in risk estimates, however, there was no significant difference in the relationship between dairy consumption and risk of MetS with study design, sex, geographical region, or criteria (*p*_difference_ > 0.5 for all comparisons). For adjustment for confounders, an inverse association was found in studies adjusted for energy intake, but the difference in the pooled RRs with energy intake adjustment was not significant (*p*_difference_ = 0.28). In addition, there was no significant difference with other adjustment factors (*p*_difference_ > 0.2 for all comparisons). Six cohort studies [22,35,38,40,41,43] were included in the dose-response analysis for total dairy consumption and incidence of MetS (Table 5). The pooled RR for 400 g/day increment of total dairy consumption was 0.71 (95% CI: 0.59–0.85, *I*^2^ = 72.4%, *p* = 0.003).

#### 3.2.3. Milk Consumption and MetS

Twenty studies investigated the association between the highest versus the lowest milk consumption and risk of MetS, and included 341,426 participants (Table 3). The pooled RR for all studies was 0.83 (95% CI: 0.78–0.89) (Table 4), with evidence of substantial heterogeneity (*I*^2^ = 72.5%, *p* < 0.001). Furthermore, in the stratified analysis by study design, we found a similar inverse association in cohort and cross-sectional studies (Table 4, Figure S1b). By sex, we found a significant inverse association in men and women, and no significant difference was found (*p*_difference_ = 0.70). By geographic region, there was a significant inverse association in America and Asia, while there was no significant association in Europe. By criteria of MetS, significant inverse associations were shown in NCEP ATP III and JIS criteria, while no significant association was found in IDF and other criteria. In the meta-regression analysis, however, the pooled RRs were not significantly different with criteria of MetS (*p*_difference_ for JIS, IDF, or other criteria versus NCEP ATP III criteria = 0.88, 0.76, and 0.98, respectively). By adjustment factors, we found no significant difference with BMI, energy intake, alcohol, smoking, or physical activity (*p*_difference_ > 0.2 for all comparisons). Five cohort studies [22,34,41,43,44] were included in the dose-response analysis for milk consumption and incidence of MetS (Table 5). A 200 g/day increment of milk consumption was associated with 15% decrease incidence of MetS (RR: 0.85; 95% CI: 0.79–0.93), with no significant heterogeneity (*I*^2^ = 51.8%, *p* = 0.08).

#### 3.2.4. Yogurt Consumption and MetS

Twelve studies investigated the association between the highest versus the lowest yogurt consumption and risk of MetS, and included 300,600 participants (Table 3). The pooled RR for all studies was 0.89 (95% CI: 0.83–0.95), with some evidence of heterogeneity (*I*^2^ = 69.1%, *p* < 0.001). By study design, we found a similar inverse association in cohort and cross-sectional studies (Table 4, Figure S1c). By geographic region, a significant inverse association was found in Asia and Europe, while there was no significant association in America. Furthermore, the heterogeneity disappeared in America (*I*^2^ = 51.1%, *p* = 0.13) and Europe (*I*^2^ = 0%, *p* = 0.65) when we stratified by the region. By MetS criteria, a significant inverse association was found in the NCEP ATP III and JIS criteria, while only one study was included in IDF and other criteria. There was no significant difference in geographic region or criteria (*p*_difference_ > 0.05 for all comparisons). Regarding adjustment for confounders, we found no significant difference with BMI, energy intake, alcohol, smoking, or physical activity (*p*_difference_ > 0.1 for all comparisons). Five cohort studies [22,41,42,43,44] were included in the dose-response analysis for yogurt consumption and incidence of MetS (Table 5). A 200 g/day increment of yogurt intake was associated with 37% decrease incidence of MetS (RR: 0.63; 95% CI: 0.53–0.75), with no significant heterogeneity (*I*^2^ = 0.3%, *p* = 0.40).

#### 3.2.5. Cheese Consumption and MetS

Eight studies investigated the association between the highest versus the lowest total dairy consumption and risk of MetS, and included 129,822 participants (Table 3). The pooled RR for all studies was 0.98 (95% CI: 0.86–1.11), with evidence of substantial heterogeneity (*I*^2^ = 85.6%, *p* < 0.001). Similarly, there was no significant association both in cohort and cross-sectional studies (Table 4, Figure S1d). Based on the results of meta-regression analyses, there was no significant difference with study design, geographic region, or MetS criteria (*p*_difference_ > 0.4 for all comparisons). By adjustment for confounders, we found a significant positive association in studies that had adjusted for alcohol drinking, and the result of meta-regression analysis showed a significant difference (*p*_difference_ = 0.02). Three cohort studies [38,41,44] were included in the dose-response analysis for cheese intake and incidence of MetS (Table 5). The pooled RR for 50 g/day increment of cheese consumption was 0.99 (95% CI: 0.73–1.35, *I*^2^ = 86.2%, *p* = 0.001).

#### 3.2.6. Publication Bias

There was no evidence of publication bias with Begg’s (*p* > 0.1 in all analysis) and Egger’s test (*p* > 0.05 in all analysis) of risk of MetS for total dairy, milk, yogurt, and cheese consumption.

## 4. Discussion

In the present meta-analysis, we found significant inverse associations with the risk of MetS for the highest vs. lowest categories of total dairy, milk, and yogurt consumption, while cheese consumption showed no significant association overall. People in the highest category of total dairy, milk, and yogurt consumption had a decreased risk of MetS of 20%, 17%, and 11%, respectively, compared with those in the lowest intake category. The results of dose–response analysis also supported the associations.

We conducted several subgroup analyses in the present study. Published previous two meta-analyses conducted a subgroup analysis depending on the study design [6,7], and one meta-analysis conducted a subgroup analysis of total dairy and milk intake [8]. In addition, the most recent meta-analysis included only prospective cohort studies, and no subgroup analysis was performed [9]. We performed an analysis stratified by study design, and found no significant difference in study design. Thus, we included both cohort and cross-sectional studies, and conducted subgroup analyses for total dairy products, milk, yogurt, and cheese consumption. Moreover, we included more recent two cohort studies (three articles) [23,24,44] and 11 cross-sectional studies (11 articles and the current results of the KNHANES data) [21,56,57,58,59,60,61,62,63,64,65] including the first large multinational studies [61]. In the present meta-analysis, we not only performed stratified analyzes by geographic region and adjustment for confounders like the previous meta-analyses [7,8] but also performed analyses stratified by sex and MetS criteria.

Some evidence of heterogeneity among the studies was found in the meta-analysis of dairy consumption and the risk of MetS. The observed heterogeneity tended to disappear when stratified by geographic region or MetS criteria. According to the region, the dairy products consumption varies greatly. Europe is the region with the highest consumption of dairy products, while the intake of dairy products in Asia is much lower than that of European and American countries [66]. The result of KNHANES data in the current study also reflects this trend. More than three-quarters of people did not consume dairy products. In addition, the composition of dairy products varies according to different regions. Europe and the United States are both countries with high consumption of dairy products, but the United States consumes a higher proportion of processed dairy products than Europe [67]. There are more than one diagnostic criteria for MetS. Although the general standards are similar, they are not the same. According to the IDF definition, for a person to be diagnosed with MetS, they must have abdominal obesity and any two of the four factors [2]. For the JIS criteria, people with any three of five risk factors are diagnosed with MetS and there are standard recommended waist circumference thresholds for abdominal obesity by region [1]. The NCEP ATP III criteria requires at least three of the five components for the diagnosis of MetS [13], and several studies combined their region standard of waist circumference thresholds and made a modified NCEP ATP III criteria. The use of different criteria for MetS may lead to heterogeneity of the study results.

In the current analysis of KNHANES data, the study showed that consumption of milk was inversely associated with the prevalence of MetS in Korean elderly, but not in adults. Compared with adults, low-fat or skimmed milk accounts for a higher proportion of the total milk intake of the elderly. The previous cohort study reported that low-fat milk intake decreased the risk of MetS in the elderly, whereas whole-fat milk intake had no significant association [41]. Moreover, in the result of additional analysis of MetS components, milk consumption was associated with low HDL-cholesterol in elderly women. The percentage of total energy from carbohydrates in elderly women was extremely high, even exceeding the KDRI standard [12], and this tendency was more serious in non-consumers. The previous study reported that high carbohydrate intake were significantly associated with low HDL-cholesterol levels in women [68]. Although there was no significant association between milk consumption and risk of MetS in adults, milk consumption was associated with hypertriglyceridemia in adult men, and a previous study supported this association [69]. Regarding the consumption of yogurt, although the yogurt intake in adult women and elderly men was inversely associated with hyperglycemia, there was not significantly associated with the prevalence of MetS. This result may be because the type of yogurt consumed in Korea is different from traditional yogurts such as Greek yogurt or non-curd yogurt in Western countries [60]. In Korea, the most popular yogurt contains a lot of sugar [60]. Several previous studies have shown that consumption of fructose may cause type 2 diabetes and cardiovascular disease [70], but there was no study to explain the effect of adding sugar to yogurt on metabolism. On the other hand, although people who consumed higher levels of yogurt had higher calcium intake than non-consumers, the difference was not as extreme as the milk consumption.

The several potential mechanisms could explain the beneficial effect of dairy product consumption such as milk and yogurt on MetS. Dairy products contain several minerals such as calcium and potassium [71]. Several previous studies showed that increased intake of dairy products or calcium could lead to the prevention of weight gain or weight regain [72,73]. Increasing the dairy products or calcium intake can impair fat absorption by forming insoluble soaps, leading to weight and fat loss [71,74]. Moreover, some studies showed that an increase of calcium in the diet leads to an increase in fecal fat excretion [73]. Calcium combines with bile acids in the intestine to inhibit their absorption, and this is associated with reduced serum low-density lipoprotein cholesterol [4,74,75]. Insufficient calcium intake increases the level of 1,25-dihydroxycholecalciferol in plasma, which increases intracellular calcium concentration in adipocytes, resulting in the inhibition of lipolysis and FA synthase gene expression, thereby leading to the expansion of triglycerides storage in adipocytes [76]. By contrast, adequate calcium intake reduces the level of 1,25-dihydroxycholecalciferol in plasma, which leads to decrease in intracellular calcium in adipocytes, thereby reducing fatty acid (FA) synthase and increasing lipolytic activity [4,76]. In addition, calcium and potassium affect MetS through their effects on blood pressure [71]. Adequate dairy intake affects blood pressure control, which may be due to the ability of calcium and potassium to reduce sodium retention through several mechanisms [77]. 1,25-Dihydroxycholecalciferol stimulates calcium influx through vitamin D receptors, thereby promoting contraction and peripheral resistance [78]. Therefore, adequate calcium consumption will reduce 1,25-dihydroxycholecalciferol and thus lower blood pressure [78]. Potassium in the diet can reduce blood pressure by inhibiting proinflammatory events in vascular smooth muscle cells, reducing platelet aggregation and renal vascular resistance [71,79].

Dairy products also contain protein, which mainly includes casein and whey proteins. Hydrolyzed whey protein can inhibit the angiotensin-converting enzyme in vitro, thereby inducing the inhibition of angiotensin II [4,71]. In addition to the blood pressure regulating effect of angiotensin II, it can also up-regulate the expression of FA synthase gene, leading to adipocyte lipogenesis [4,80,81]. Thus, whey protein reduces endogenous fat, resulting in decreased plasma triglycerides, total cholesterol, and low-density lipoprotein (LDL)-cholesterol [71,82]. Dairy products contain also more than 400 different identified FAs and FA derivatives [5]. Among them, saturated FAs are the predominant FAs type in milk fat, accounting for about 64–73% of total FAs [5]. Butyric acid, one of the representative short-chain FAs included in dairy products, can induce positive changes in systemic metabolism [5]. Animal studies have shown that butyrate provided by a high-fat diet mitigates weight gain [83] and improves insulin sensitivity [84]. Similarly, the consumption of medium-chain FAs and long-chain FAs can also induce positive changes in metabolism [5]. Caprylic acid, a medium-chain FA, can beneficially influence glucose and energy homeostasis [5]. A diet rich in capric acid, another medium-chain FA, can lower total cholesterol and triglycerides [85,86]. Some research suggests that stearic acid (long-chain FA) consumption can have a positive effect on whole-body energy metabolism [5]. However, a previous study reported that intake of long-chain FA had an increased risk of coronary heart disease [87].

The nutritional ingredients of dairy products vary according to each dairy product type. Yogurt is rich in probiotic bacteria that can beneficially improve the gut microbiota, which has a positive effect on weight control [88]. In addition, a previous meta-analysis of randomized trials showed that probiotic consumption may prevent and control hyperglycemia [89]. Cheese is a high-fat dairy product, and whey protein is lost during the cheese-making process. These differences may cause different effects between milk or yogurt and cheese. In addition, one cohort study reported cheese consumption was associated with an increased risk of MetS and explained that cheese has higher phosphorus content and energy density, and sodium than other dairy products [41]. However, a previous randomized trial showed that a high intake of cheese did not increase total or LDL cholesterol level and it was related to a slight decrease in cholesterol level in those participants who had MetS at baseline, compared with the control group of low cheese intake [90]. In the current study, cheese consumption was not significantly associated with the risk of MetS, and showed a slightly higher risk of MetS when limited to studies that had adjusted for alcohol consumption. Interestingly, all previous studies that cheese consumption had a positive effect on MetS have not adjusted for alcohol consumption [38,53,61], and a previous meta-analysis presented lower risk of MetS in light alcohol drinkers [91]. Thus, there is a lack of comprehensive explanation of the association between cheese intake and MetS, and more studies on cheese consumption are needed.

There are some strengths of the present meta-analysis. We performed several subgroup analyses with different types of dairy product. To the best of our knowledge, this is the first meta-analysis to assess the association between cheese consumption and MetS risk, and different types of dairy products have been individually stratified by sex, MetS criteria, and geographical region. The current study included the most recent prospective cohort and cross-sectional data, and the largest number of study subjects. Many studies included in the meta-analysis have adjusted for confounding factors such as age, sex, energy intake, alcohol consumption, smoking, or physical activity. In addition, the present study assessed a linear dose-response meta-analysis between dairy products consumption, such as total dairy, milk, yogurt, and cheese, and MetS incidence.

The present meta-analysis also has several limitations. First, this meta-analysis was based on observational studies and, therefore, possible residual or unmeasured confounding factors cannot be excluded from the results. Second, most of the studies included in current meta-analysis used self-reported food frequency questionnaires (FFQs) to assess the consumption of dairy products, and therefore, it is necessary to consider the possible measurement errors when recording information during the assessment. Third, the cut-offs for the highest and lowest consumption categories varied among the studies. To address this limitation, we conducted a dose-response meta-analysis as well. Fourth, in the KNHANES study, we used dietary data collected from 24 h dietary recall in order to include both adults and elderly, and thus, it may reflect the short-term intake of the participants rather than their usual intake.

## 5. Conclusions

In conclusion, the results of this meta-analysis of prospective cohort studies and cross-sectional studies suggest that total dairy products, milk, and yogurt consumption is associated with reduced risk of MetS, while cheese consumption is not significantly associated with the risk of MetS. The use of different diagnostic criteria for MetS and different geographical regions may have affected the heterogeneity of the results. Although our findings may provide deeper insights into the consumption of dairy products, further well-designed prospective cohort studies on different types of dairy products and different types of study populations are warranted. In addition, randomized clinical trials should be conducted to examine the association between different types of dairy products consumption and the risk of MetS to provide definitive evidence.

## Figures and Tables

**Table 1:** Multivariable-adjusted odds ratio (ORs) and 95% confidence intervals (CIs) for metabolic syndrome according to milk consumption in Korean adult and elderly population.

	Milk Consumption (Servings/Day)			*p* Trend ^1^
	0	0< to <1	≥1	
All adults (4005 cases/18,206 subjects)				
No. of cases/subjects	3175/13,664	313/1701	517/2841	
Age-adjusted OR (95%CI)	1.0 (reference)	0.76 (0.65–0.88)	0.78 (0.70–0.89)	<0.001
Multivariable-adjusted OR (95%CI) ^2^	1.0 (reference)	1.01 (0.85–1.20)	0.91 (0.78–1.06)	0.246
Men (2208 cases/7488 subjects)				
No. of cases/subjects	1775/5855	149/519	284/1114	
Age-adjusted OR (95%CI)	1.0 (reference)	0.92 (0.74–1.15)	0.81 (0.69–0.96)	0.013
Multivariable-adjusted OR (95%CI) ^2^	1.0 (reference)	1.03 (0.81–1.32)	0.87 (0.71–1.06)	0.204
Women (1797 cases/10,718 subjects)				
No. of cases/subjects	1400/7809	164/1182	233/1727	
Age-adjusted OR (95%CI)	1.0 (reference)	0.76 (0.62–0.93)	0.80 (0.67–0.96)	0.002
Multivariable-adjusted OR (95%CI) ^2^	1.0 (reference)	0.96 (0.76–1.20)	0.95 (0.77–1.18)	0.608
All elderly people (2320 cases/5113 subjects)				
No. of cases/subjects	1941/4196	175/391	204/526	
Age-adjusted OR (95%CI)	1.0 (reference)	0.97 (0.76–1.23)	0.73 (0.59–0.92)	0.013
Multivariable-adjusted OR (95%CI) ^2^	1.0 (reference)	0.95 (0.73–1.24)	0.74 (0.57–0.96)	0.029
Men (859 cases/2200 subjects)				
No. of cases/subjects	737/1862	56/136	66/202	
Age-adjusted OR (95%CI)	1.0 (reference)	1.04 (0.69–1.55)	0.73 (0.51–1.05)	0.129
Multivariable-adjusted OR (95%CI) ^2^	1.0 (reference)	0.97 (0.61–1.55)	0.83 (0.56–1.23)	0.359
Women (1461 cases/2913 subjects)				
No. of cases/subjects	1204/2334	119/255	138/324	
Age-adjusted OR (95%CI)	1.0 (reference)	0.86 (0.63–1.16)	0.69 (0.52–0.92)	0.010
Multivariable-adjusted OR (95%CI) ^2^	1.0 (reference)	0.93 (0.67–1.28)	0.72 (0.52–0.99)	0.058

^1^*p* value were obtained from a regression model using the PROC SURVEYLOGISTIC procedure. ^2^ Adjusted for age (continuous), sex, body mass index (BMI, continuous), education (≤middle school, high school, or ≥college), household income (lowest, lower middle, upper middle, or highest), smoking (non-smoker, former smoker, or current smoker), alcohol intake (never/rarely, 1–4/month, or ≥2/week), physical activity (yes or no), and total energy (continuous).

**Table 2:** Multivariable-adjusted odds ratio (ORs) and 95% confidence intervals (CIs) for metabolic syndrome according to yogurt consumption in Korean adult and elderly population.

	Yogurt Consumption (Servings/Day)			*p* Trend ^1^
	0	0< to <1	≥1	
All adults (4005 cases/18,206 subjects)				
No. of cases/subjects	3554/15,797	220/1161	231/1248	
Age-adjusted OR (95%CI)	1.0 (reference)	0.74 (0.61–0.89)	0.74 (0.62–0.88)	<0.001
Multivariable-adjusted OR (95%CI) ^2^	1.0 (reference)	0.94 (0.75–1.17)	0.84 (0.70–1.02)	0.065
Men (2208 cases/7488 subjects)				
No. of cases/subjects	1999/6665	90/353	119/470	
Age-adjusted OR (95%CI)	1.0 (reference)	0.83 (0.62–1.11)	0.75 (0.58–0.96)	0.013
Multivariable-adjusted OR (95%CI) ^2^	1.0 (reference)	0.97 (0.69–1.35)	0.83 (0.63–1.08)	0.158
Women (1797 cases/10,718 subjects)				
No. of cases/subjects	1555/9132	130/808	112/778	
Age-adjusted OR (95%CI)	1.0 (reference)	0.78 (0.60–1.00)	0.76 (0.60–0.96)	0.008
Multivariable-adjusted OR (95%CI) ^2^	1.0 (reference)	0.88 (0.66–1.17)	0.87 (0.67–1.12)	0.200
All elderly people (2320 cases/5113 subjects)				
No. of cases/subjects	2000/4371	187/435	133/307	
Age-adjusted OR (95%CI)	1.0 (reference)	0.85 (0.67–1.08)	0.86 (0.65–1.12)	0.155
Multivariable-adjusted OR (95%CI) ^2^	1.0 (reference)	0.80 (0.60–1.07)	0.91 (0.68–1.21)	0.296
Men (859 cases/2200 subjects)				
No. of cases/subjects	751/1918	63/168	45/114	
Age-adjusted OR (95%CI)	1.0 (reference)	0.82 (0.56–1.19)	0.92 (0.59–1.41)	0.512
Multivariable-adjusted OR (95%CI) ^2^	1.0 (reference)	0.78 (0.51–1.21)	0.92 (0.59–1.44)	0.507
Women (1461 cases/2913 subjects)				
No. of cases/subjects	1249/2453	124/267	88/193	
Age-adjusted OR (95%CI)	1.0 (reference)	0.84 (0.63–1.14)	0.77 (0.55–1.09)	0.091
Multivariable-adjusted OR (95%CI) ^2^	1.0 (reference)	0.81 (0.57–1.15)	0.89 (0.62–1.28)	0.353

^1^*p* value were obtained from a regression model using the PROC SURVEYLOGISTIC procedure. ^2^ Adjusted for age (continuous), sex, BMI (continuous), education (≤middle school, high school, or ≥college), household income (lowest, lower middle, upper middle, or highest), smoking (non-smoker, former smoker, or current smoker), alcohol intake (never/rarely, 1–4/month, or ≥2/week), physical activity (yes or no), and total energy (continuous).

**Table 3:** Characteristics of prospective cohort/cross-sectional studies included in the meta-analysis of dairy product intake and metabolic syndrome.

First Author (Year)	Country (Study Name)	Study Design	Age (Years)	Subjects	Criteria for Metabolic Syndrome	Exposure Category	Adjustment Factors
Pereira (2002) [22]	USA (Coronary Artery Risk Development in Young Adults study, CARDIA)	Cohort	18–30	467/3157	≥2 of the 4 components: abnormal glucose homeostasis, obesity, elevated BP, and dyslipidemia.	Dairy products 0–<10 (ref.), 10–<16, 16–<24, 24–<35, ≥35 times/week Milk 1 daily increment Yogurt 1 daily increment	Age, sex, BMI, race, calorie intake/day, study center, education, smoking, alcohol, PA, vitamin supplement, polyunsaturated fat, caffeine, fiber/1000 calories, whole and refined grains, meat, fruit, vegetables, soda, magnesium, Ca and vitamin D
Damiăo (2006) [34]	Brazil	Cohort	40–79	57/151	NCEP ATP III	Milk 12.4 (ref.), 141.7, 223.7 g/day	Age, sex, smoking, PA, education, alcohol, total energy intake
Lutsey (2008) [35]	USA (Atherosclerosis Risk in Communities study, ARIC)	Cohort	45–64	3782/9514	American Heart Association guidelines	Dairy products 0.28 (ref.), 0.93, 1.29, 1.94, 3.30 servings/day	Age, sex, race, education, smoking, center, total calories, PA, pack-years, meat, dairy, vegetables, fruits, and whole and refined grains
Snijder (2008) [36]	Netherlands (Hoorn study)	Cohort	50–75	215/1124	NCEP ATP III	Dairy products Quartile (Q) 1(ref.), Q4	Age, sex, smoking, alcohol, total energy, PA
Duffey (2010) [37]	USA (Coronary Artery Risk Development in Young Adults study, CARDIA)	Cohort	18–30	459/3596	NCEP ATP III	Whole fat milk Moving from 1 quartile to the next	Age, race, sex, CARDIA exam center, weight, smoking, total PA, energy from food, the 3 other beverages, and alcohol
Fumeron (2011) [38]	France (Epidemiological Study on the Insulin Resistance Syndrome, DESIR)	Cohort	30–65	452/3435	NCEP ATP III IDF	Dairy products Per a change from 1 category to the next. Cheese Per a change from 1 category to the next.	Age, sex, smoking, total fat intake, PA, BMI
Lin (2013) [39]	Taiwan	Cohort	≥65	206/888	NCEP ATP III	Milk No (ref.), yes	Age, sex, smoking, alcohol, serum creatinine, uric acid, ALT, urine protein, initial MetS score, exercise, teeth brushing, vegetable
Louie (2013) [40]	Australia (Blue Mountains Eye Study, BMES)	Cohort	≥49	155/1807	IDF	Dairy products 0.5 (ref.), 1.2, 1.8, 3.1 servings/day	Age, sex, smoking, PA, dietary glycemic load, fibre from vegetables, family history, total energy, Ca
Babio (2015) [41]	Spain (Prevenci ‘on con Dieta Mediterr’anea, PREDIMED)	Cohort	55–80	930/1868	JIS	Dairy products 207 (ref.), 354, 577 g/day Milk 120 (ref.), 222, 462 g/day Yogurt 7 (ref.), 70, 127 g/day Cheese 11 (ref.), 28, 51 g/day	Age, sex, intervention group, BMI, leisure time PA, smoking, use of hypoglycemic, antihypertensive, hypolipidemic, insulin treatment at baseline, vegetables, fruit, legumes, cereals, red meat, fish, nuts, cookies, olive oil, alcohol, prevalence of metabolic syndrome components at baseline.
Sayón-Orea (2015) [42]	Spain (Seguimiento Universidad de Navarra, SUN)	Cohort	20–90	306/8063	JIS	Yogurt 0–250 (ref.), >250–<875, ≥875 g/week	Age, sex, smoking, alcohol, baseline weight, total energy, red meat, soft drinks, fast food, french fries, mediterranean diet, PA, sedentary behavior, hours sitting, snacking between meals, following special diet
Kim (2017) [43]	Korea (Korean Genome and Epidemiology Study, KoGES)	Cohort	40–69	2103/5510	NCEP ATP III	Dairy products None (ref.), <1, 1–<4, 4–≤7, >7 servings/week Milk None (ref.), <1, 1–<4, 4–≤7, >7 servings/week Yogurt None (ref.), <1, 1≤ to <4, ≥4 servings/week	Age, sex, BMI, smoking, alcohol, residential location, educational, household income, PA, energy, energy-adjusted Ca and fibre
Beydoun (2018) [44]	USA (Healthy Aging in Neighborhoods of Diversity across the Life Span, HANDLS)	Cohort	30–64	173/1371	NCEP ATP III	Milk Per cup equivalent Yogurt Per fl oz equivalent Cheese Per oz equivalent	Age, sex, race, smoking, alcohol, socio-economic status, energy intake at baseline, current drug use and self-rated health, energy intake, total fruit, deep yellow vegetables, dark green vegetables, non-whole grains, legumes, whole grains, nuts/seeds, soya, total meat/poultry/fish, eggs, discretionary solid fat, discretionary oils, added sugars and mg of caffeine.
Cheraghi (2018) [24]	Iran (Tehran Lipid and Glucose Study, TLGS)	Cohort	≥20	590/3616	JIS	Whole fat milk None (ref.), 1 cup/day Yogurt None (ref.), 1/2 cup/day Cheese None (ref.), 1 oz/day	Age, sex, cancer history, hospitalisation status, education, BMI, marital status, smoking, calories, 95 foods and 12 nutrients
Mirmiran (2020) [23]	Iran (Tehran Lipid and Glucose Study, TLGS)	Cohort	≥19	368/1114	JIS	Dairy products Per 1 standard deviation	Age, sex, academic educations, baseline BMI, BMI-change, and energy intakes.
Mennen (2000) [45]	France(Data from an Epidemiological Study on the Insulin Resistance syndrome, DESIR)	Cross-sectional	30–64	1601/4976	≥2 of the 4 components: serum triglycerides, diastolic BP or fasting glucose in the upper quartile of the distribution or HDL cholesterol in the lowest quartile (Quartiles were gender-specific).	Dairy products ≤1 (ref.), >1–4, >4 portion/day	Age, energy intake, waist- hip ratio
Azadbakht (2005) [46]	Iran (Tehran Lipid and Glucose Study, TLGS)	Cross-sectional	18–74	827	NCEP ATP III	Dairy products <1.7 (ref.), 1.7–2.3, 2.3–3.1, ≥3.1 servings/day	Age, BMI, total energy, percent of energy from fat, smoking, use of BP and estrogen medication, PA, food group, Ca, and protein intake
Lawlor (2005) [17]	UK (British Women’s Health Study)	Cross-sectional	60–79	4024	WHO	Milk Non milk drinker(ref), milk drinker	Age
Liu (2005) [47]	USA (Women’s Health Study)	Cross-sectional	≥45	10,066	NCEP ATP III	Dairy products <0.91 (ref.), 0.91–1.41, 1.42–1.99, 2.00–3.00, >3.00 servings/day Milk <0.13 (ref.), 0.13–0.43, 0.44–0.93, 0.94–1.07, >1.08 servings/day	Age, smoking, alcohol, total calorie intake, and randomized treatment assignment, exercise, total calories, multivitamin, family history, dietary intakes of total fat, cholesterol, protein, and glycemic load
Elwood (2007) [48]	UK (Caerphilly Cohort Study)	Cross-sectional	45–59	2375	WHO	Milk Little or none (ref.), <1/2, 1/2–1, >1 pint	Age, social class and smoking
Ruidavets (2007) [49]	France	Cross-sectional	45–64	912	NCEP ATP III	Dairy products Q1 (ref.), Q2, Q3, Q4, Q5	Age, centre, smoking, alcohol, PA, energy intake, education, drugs for hypertension and dyslipidaemia, dieting, and diet quality index
Beydoun (2008) [25]	USA(National Health and Nutrition Examination Survey, NHANES)	Cross-sectional	≥18	4519	NCEP ATP III	Dairy products Each daily servings Whole milk per 100 g Yogurt Each daily servings Cheese Each daily servings	Age, sex, ethnicity, socioeconomic status, energy intake, PA, alcohol, total fruit, deep yellow vegetables, dark green vegetables, non-whole grains, whole grains, legumes, nuts/seeds, soy, total meat/poultry/fish, eggs, discretionary solid fat, discretionary oils, added sugars, and mg of caffeine.
Kwon (2010) [50]	Korea (KNHANES III)	Cross-sectional	≥19	1066/4890	NCEP ATP III	Milk rarely (ref.), ≤1/week, 2–6/week, ≥1/day	Age, sex, BMI, education, smoking, PA, alcohol, energy, and fiber intake
Jung (2011) [18]	Korea (Bundang Jesaeng General Hospital, BJGH)	Cross-sectional	30–59	142/596	NCEP ATP III	Dairy products Q1 (ref.), Q2, Q3, Q4	Age, sex, energy intake
Mosley (2013) [19]	Mexico (2009 UP AMIGOS cohort)	Cross-sectional	18–25	339	JIS	Dairy products <3 (ref.), ≥3 servings/day Whole milk <7 (ref.), ≥7 servings/week Cheese <7 (ref.), ≥7 servings/week	Age, sex, total calorie, family history, and PA
Kim (2013) [51]	Korea (KNHANES V-1)	Cross-sectional	≥19	4862	JIS	Milk None or rarely (ref.), ≤2–3/month, ≤4–6/week, ≥once/day Yogurt None or rarely (ref.), ≤2–3/month, ≤4–6/week, ≥once/day	Age, sex, education, income, smoking, BMI, alcohol, PA, energy, fat, Ca, and fibre intake
Sadeghi (2014) [53]	Iran (Isfahan Healthy Heart Program, IHHP)	Cross-sectional	37.84, 39.08	1752	3 or more factor: FBS > 126 mg/dl or waist > 102 cm for men and >85 cm for women or TG > 150 mg/dl or HLD < 40 mg/dl for men and <50 mg/dl for women or systolic BP > 130 mmHg and diastolic > 85 mmHg.	Cheese <7 (ref.), ≥7 times/week	Age, sex, dietary intake, PA, BMI
Kai (2014) [52]	France (The 2005–2007 MONA LISA multicentre cross-sectional population survey)	Cross-sectional	35–64	3078	JIS	Dairy products 0–13.2 (ref.), 13.3–23.1, 23.2–36.3, 36.4–266.0 g/1000 kJ	Age, sex, region, education, PA, alcohol, smoking, diet, total energy intake and Programme National Nutrition Sante’—Global Score
Martins (2015) [54]	Brazil (Perinatal Health in Ribeirao Preto, Sao Paulo, Brazil)	Cross-sectional	23–25	242/2031	IDF JIS	Dairy products 0.0–0.6 (ref.), 0.7–1.2, 1.3–1.7, 1.8–2.6, 2.7–14.2 portions/d	Age, sex, smoking, alcohol, PA, calorie intake, schooling and marital status, carbohydrate, protein intake, fat, bread and cereal, vegetables, fruits, meats, sugar and fats, Ca
Strand (2015) [20]	China (North China Urban Middle-Aged Population)	Cross-sectional	44, 48, 52	793	NCEP ATP III	Milk Rarely (ref.), sometimes, often	Age, sex, education, exercise, alcohol, smoking, chronic disease knowledge score, family history
Drehmer (2016) [55]	Brazil (Brazilian Longitudinal study of Adult Health, ELSA-Brasil)	Cross-sectional	35–74	9835	JIS	Dairy products <1 (ref.), 1–2, >2–4, >4 servings/day	Age, sex, race, alcohol, PA, education, occupational status, family income, study center, menopausal status, family history, smoking, and calorie intake, nondairy food groups
Falahi (2016) [56]	Iran	Cross-sectional	18–75	282/973	JIS	Yogurt Across mean consumption	Age, sex, smoking, PA, history of diabetes and heart disease, BMI, energy intake milk and cheese intake
Shin (2017) [60]	Korea (the Health Examinees study, HEXA)	Cross-sectional	40–69	34,039/130,420	NCEP ATP III	Milk M: Non or Rarely (ref.), ≤2/week, 3–6/week, ≥1/day F: Non or Rarely (ref.), ≤2/week, 3–6/week, 1/day, ≥2/day Yogurt The lowest (ref.), the highest	Age, BMI, recruitment site, education, smoking, alcohol, regular exercisers, and total energy intake.
Guo (2017) [57]	China	Cross-sectional	≥18	4305/15,020	JIS	Milk no or <0.5 (ref.), 0.5–1.5, ≥1.5 L/week	Age, education, minority, vegetables, fresh meat, drinking and smoking
Kim (2017) [58]	Korea (KNHANES IV-2,3, V-1,2)	Cross-sectional	30–64	3143/11,029	NCEP ATP III	Milk Q1 (ref.), Q2, Q3	Age, sex, total energy intake, diet modification, and education level
Mahanta (2017) [59]	India	Cross-sectional	20–60	1606/3372	NCEP ATP III	Dairy products <3 (ref.), ≥3 times/week	Age, religion, education, occupation, car, motorcycle, television, other land/property, computer, family history (hypertension, diabetes), tobacco user, consumed alcohol, financial stress, felt stress in last year, active at work, meat, fish, egg, high energy food, desserts/sweet, nuts/seeds, and past 12 months, was ever you felt sad, blue or depressed for 2 weeks or more in a row
Chang (2019) [21]	Taiwan	Cross-sectional	≥20	366/1066	NCEP ATP III	Dairy products Seldom(ref.), often	Age, education, marital status and employment
Bhavadharini (2020) [61]	Multinational (Prospective Urban Rural Epidemiological Study, PURE)	Cross-sectional	35–70	112,922	JIS	Dairy products 0(ref.), <1, 1–2, >2 servings/day Milk 0(ref.), 0–0.5, 0.5–1, >1 servings/day Yogurt 0(ref.), 0–0.5, 0.5–1, >1 servings/day Cheese 0(ref.), 0–0.5, 0.5–1, >1 servings/day	Age, sex, smoking, energy intake, education, location, PA, fruit and vegetable intake, percent energy from carbohydrate, and study center as random effect
Pasdar (2020) [64]	Iran	Cross-sectional	30–65	52/112	IDF	Dairy products <3 (ref.), 3–5, >5 times/day	Age, BMI, and PA
Hidayat (2020) [62]	China	Cross-sectional	≥18	2387/5149	JIS	Milk Non-consumer (ref.), consumer	Age, sex, smoking, alcohol, BMI, PA, education, television watching duration, sleep duration, and consumption of fish, red meat, poultry, vegetables, fruits, nut, soya and salted vegetables
Mohammadifard (2020) [63]	Iran (Isfahan Healthy Heart Program, IHHP)	Cross-sectional	≥19	9553	NCEP ATP III	Dairy products <12 (ref.), 12–16, >16–21, >21 times/week	Age, sex, urbanization, educational level education, BMI, PA, history of CVD, and dietary factors
Jin (2020) [65]	Korea (KNHANES VI, Ⅶ)	Cross-sectional	≥19	6325/23,319	NCEP ATP III	Dairy products 0 (ref.), 0< to <1, ≥1 serving/day	Age, sex, smoking, alcohol, BMI, education, household income, PA, and total energy
KNHANES ^1^	Korea (KNHANES VI, Ⅶ)	Cross-sectional	≥19	6325/23,319	NCEP ATP III	Milk 0 (ref.), 0< to <1, ≥1 serving/day Yogurt 0 (ref.), 0< to <1, ≥1 serving/day	Age, sex, smoking, alcohol, BMI, education, household income, PA, and total energy

Abbreviations: BP, blood pressure; BMI, body mass index; PA, physical activity; NCEP ATP III, National Cholesterol Education Program Adult Treatment Panel III; IDF, International Diabetes Federation; ALT, alanine aminotransferase; JIS, Joint Interim Statement; WHO, World Health Organization; KNHANES, Korea National Health and Nutrition Examination Survey; M, male; F, female; CVD, Cardiovascular Disease. ^1^ Data of the KNHANES analysis from the current paper.

**Table 4:** Subgroup-specific pooled of pooled relative risks for dairy consumption and risk of metabolic syndrome.

Subgroups	No. of Studies	Relative Risk (95% CI)	*p*_difference_
Dairy	22	0.80 (0.72–0.88)	
Study design			
Cohort	8	0.75 (0.65–0.87)	0.53
Cross-sectional	16	0.82 (0.72–0.92)	
Sex			
Men	8	0.77 (0.62–0.95)	0.66
Women	7	0.72 (0.59–0.88)	
Geographical region			
America	7	0.83 (0.69–0.99)	
Asia	8	0.78 (0.63–0.96)	0.85 ^1^
Europe	5	0.85 (0.78–0.93)	0.99 ^1^
Oceania	1	0.62 (0.24–1.61)	0.70 ^1^
Criteria			
NCEP ATP III	12	0.82 (0.71–0.94)	
JIS	7	0.77 (0.72–0.83)	0.62 ^2^
IDF	4	0.73 (0.43–1.24)	0.87 ^2^
Other	3	0.76 (0.60–0.95)	0.55 ^2^
Adjustment for confounders			
BMI			
Yes	8	0.75 (0.66–0.86)	0.41
No	14	0.84 (0.72–0.97)	
Energy intake			
Yes	16	0.76 (0.69–0.85)	0.28
No	6	0.90 (0.69–1.16)	
Alcohol			
Yes	12	0.84 (0.72–0.99)	0.28
No	10	0.77 (0.70–0.86)	
Smoking			
Yes	15	0.81 (0.73–0.90)	0.37
No	7	0.71 (0.56–0.91)	
Physical activity			
Yes	19	0.82 (0.74–0.91)	0.31
No	3	0.66 (0.50–0.87)	
Milk	20	0.83 (0.78–0.89)	
Study design			
Cohort	7	0.83 (0.72–0.97)	0.94
Cross-sectional	13	0.83 (0.77–0.90)	
Sex			
Men	7	0.83 (0.75–0.92)	0.70
Women	7	0.79 (0.69–0.90)	
Geographical region			
America	6	0.86 (0.78–0.95)	
Asia	10	0.80 (0.72–0.89)	0.65 ^3^
Europe	3	0.87 (0.45–1.71)	0.72 ^3^
Criteria			
NCEP ATP III	11	0.84 (0.77–0.92)	
JIS	7	0.84 (0.77–0.93)	0.88 ^4^
IDF	1	0.79 (0.59–1.07)	0.76 ^4^
Other	3	0.83 (0.41–1.67)	0.98 ^4^
Adjustment for confounders			
BMI			
Yes	8	0.81 (0.73–0.89)	0.53
No	12	0.86 (0.78–0.95)	
Energy intake			
Yes	13	0.83 (0.78–0.89)	0.89
No	7	0.83 (0.66–1.05)	
Alcohol			
Yes	14	0.82 (0.75–0.88)	0.47
No	6	0.88 (0.75–1.04)	
Smoking			
Yes	16	0.81 (0.76–0.88)	0.27
No	4	0.95 (0.75–1.20)	
Physical activity			
Yes	14	0.83 (0.77–0.90)	0.88
No	6	0.84 (0.71–0.99)	
Yogurt	12	0.89 (0.83–0.95)	
Study design			
Cohort	6	0.84 (0.71–0.98)	0.27
Cross-sectional	6	0.93 (0.87–0.99)	
Sex			
Men	4	0.86 (0.72–1.02)	0.71
Women	4	0.91 (0.81–1.02)	
Geographical region			
America	3	0.71 (0.42–1.22)	
Asia	6	0.91 (0.84–0.998)	0.70 ^5^
Europe	2	0.78 (0.67–0.91)	0.70 ^5^
Criteria			
NCEP ATP III	4	0.81 (0.68–0.97)	
JIS	6	0.89 (0.81–0.98)	0.40 ^6^
IDF	1	1.00 (0.93–1.06)	0.09 ^6^
Other	1	0.58 (0.20–1.67)	0.56 ^6^
Adjustment for confounders			
BMI			
Yes	8	0.89 (0.82–0.97)	0.84
No	4	0.89 (0.81–0.97)	
Energy intake			
Yes	11	0.90 (0.85–0.97)	0.32
No	1	0.77 (0.65–0.91)	
Alcohol			
Yes	9	0.86 (0.77–0.95)	0.30
No	3	0.94 (0.85–1.03)	
Smoking			
Yes	11	0.90 (0.84–0.96)	0.12
No	1	0.42 (0.18–0.99)	
Physical activity			
Yes	10	0.87 (0.81–0.94)	0.27
No	2	0.96 (0.87–1.06)	
Cheese	8	0.98 (0.86–1.11)	
Study design			
Cohort	4	1.03 (0.87–1.22)	0.43
Cross-sectional	4	0.91 (0.74–1.14)	
Geographical region			
America	3	1.07 (0.93–1.25)	
Asia	2	0.92 (0.71–1.20)	0.66 ^7^
Europe	2	1.03 (0.65–1.64)	0.996 ^7^
Criteria			
NCEP ATP III	3	1.00 (0.83–1.20)	
JIS	4	1.01 (0.79–1.29)	0.91 ^8^
IDF	1	0.88 (0.77–1.00)	0.61 ^8^
Other	1	0.81 (0.70–0.93)	0.42 ^8^
Adjustment for confounders			
BMI			
Yes	4	0.98 (0.78–1.21)	0.97
No	4	0.99 (0.82–1.18)	
Energy intake			
Yes	5	1.00 (0.87–1.16)	0.75
No	3	0.95 (0.71–1.27)	
Alcohol			
Yes	3	1.15 (1.01–1.30)	0.02
No	5	0.87 (0.79–0.96)	
Smoking			
Yes	5	0.99 (0.86–1.14)	0.75
No	3	0.92 (0.66–1.29)	
Physical activity			
Yes	6	0.95 (0.80–1.14)	0.63
No	2	1.03 (0.95–1.12)	

Abbreviations: NCEP ATP III, National Cholesterol Education Program Adult Treatment Panel III; IDF, International Diabetes Federation; JIS, Joint Interim Statement. ^1^ *p* value for difference in RRs of dairy consumption for Asia versus America, Europe versus America, and Oceania versus America. ^2^ *p* value for difference in RRs of dairy consumption for JIS criteria versus NCEP ATP III criteria, IDF criteria versus NCEP ATP III criteria, and other criteria versus NCEP ATP III criteria. ^3^ *p* value for difference in RRs of milk consumption for Asia versus America and Europe versus America. ^4^ *p* value for difference in RRs of milk consumption for JIS criteria versus NCEP ATP III criteria, IDF criteria versus NCEP ATP III criteria, and other criteria versus NCEP ATP III criteria. ^5^ *p* value for difference in RRs of yogurt consumption for Asia versus America and Europe versus America. ^6^ *p* value for difference in RRs of yogurt consumption for JIS criteria versus NCEP ATP III criteria, IDF criteria versus NCEP ATP III criteria, and other criteria versus NCEP ATP III criteria. ^7^ *p* value for difference in RRs of cheese consumption for Asia versus America and Europe versus America. ^8^ *p* value for difference in RRs of cheese consumption for JIS criteria versus NCEP ATP III criteria, IDF criteria versus NCEP ATP III criteria, and other criteria versus NCEP ATP III criteria. Abbreviations: NCEP ATP III, National Cholesterol Education Program Adult Treatment Panel III; JIS, Joint Interim Statement IDF; International Diabetes Federation.

**Table 5:** Pooled RRs of dairy consumption and metabolic syndrome incidence from dose–response meta-analysis.

	No of Studies	Dose	Relative Risk (95% CI)	Heterogeneity
Total airy	6	400 g/day	0.71 (0.59–0.85)	*I*^2^ = 72.4%, *p* = 0.003
Milk	5	200 g/day	0.85 (0.79–0.93)	*I*^2^ = 51.8%, *p* = 0.08
Yogurt	5	200 g/day	0.63 (0.53–0.75)	*I*^2^ = 0.3%, *p* = 0.40
Cheese	3	50 g/day	0.99 (0.73–1.35)	*I*^2^ = 86.2%, *p* = 0.001

## Data Availability

Data is contained within the article or supplementary material.

## Supplementary Materials

The following are available online at https://www.mdpi.com/article/10.3390/nu13051574/s1, Table S1: General characteristics of the study participants according to milk and yogurt consumption in the Korean adult and elderly population, Table S2: Gender-specific general characteristics according to milk consumption in the Korean adult and elderly population, Table S3: Gender-specific general characteristics according to yogurt consumption in the Korean adult and elderly population, Table S4: Multivariable-adjusted odds ratio (ORs) and 95% confidence intervals (CIs) for metabolic syndrome components according to milk consumption in Korean adult and elderly population, Table S5: Multivariable-adjusted odds ratio (ORs) and 95% confidence intervals (CIs) for metabolic syndrome components according to yogurt consumption in Korean adult and elderly population. Figure S1: Forest plot of the observational studies for risk of metabolic syndrome.
