# Supplementary material for: Dairy Consumption and Risk of Metabolic Syndrome: Results from Korean Population and Meta-Analysis
Source: Nutrients. 2021 May 8;13(5):1574. doi: 10.3390/nu13051574 (PMC8151357; doi:10.3390/nu13051574)
Supplement: Supplementary file 1 [file nutrients-13-01574-s001.zip › nutrients-1191646-supplementary.pdf]

Supplementary Table S1. General characteristics of the study participants according to milk and yogurt consumption in the Korean adult and elderly population.

		Milk consumption (servings/day)			*P* value[1]	Yogurt consuption (servings/day)			*P* value[1]
		0	0<to<1	≥1		0	0<to<1	≥1	
Adults (19-64 years)		13664	1701	2841		15797	1161	1248	
Age (years)[2]		41.6±0.1	41.4±0.4	39.6±0.3	<0.001	41.0±0.1	43.1±0.5	42.6±0.4	<0.001
BMI (kg/m²)[2]		23.9±0.0	23.4±0.1	23.6±0.1	<0.001	23.8±0.0	23.4±0.1	23.6±0.1	0.002
Sex, n(%)	Men	5855(52.8)	519(39.7)	1114(49.2)	<0.001	6665(52.0)	353(39.6)	470(48.9)	<0.001
	Women	7809(47.2)	1182(60.3)	1727(50.8)		9132(48.0)	808(60.4)	778(51.1)	
Education, n(%)	≤Middle school	2588(14.7)	230(10.0)	356(9.7)	<0.001	2764(13.6)	223(14.4)	187(11.4)	0.025
	High school	5284(40.7)	587(35.9)	1097(40.7)		6070(40.5)	438(39.5)	460(37.7)	
	≥College	5784(44.6)	883(54.2)	1388(49.6)		6954(45.9)	500(46.1)	601(50.9)	
Household income, n(%)	Lowest	1293(9.0)	112(6.2)	192(6.7)	<0.001	1433(8.7)	86(6.8)	78(5.9)	<0.001
	Lower middle	3329(23.7)	360(20.3)	654(22.3)		3803(23.6)	271(21.5)	269(19.8)	
	Upper middle	4314(32.0)	543(32.6)	913(33.3)		5055(32.4)	361(31.5)	354(29.9)	
	Highest	4697(35.3)	683(40.9)	1076(37.8)		5474(35.3)	439(40.2)	543(44.3)	
Smoking, n(%)	Non-smoker	8329(55.2)	1214(65.6)	1863(60.7)	<0.001	9714(56.0)	839(66.0)	853(62.9)	<0.001
	Former smoker	2442(19.5)	267(18.5)	504(19.5)		2835(19.6)	159(15.8)	219(19.6)	
	Current smoker	2893(25.3)	220(15.9)	474(19.8)		3248(24.4)	163(18.2)	176(17.5)	
Alcohol consumption, n(%)	Never/ rarely	5375(35.5)	780(42.4)	1178(37.6)	<0.001	6221(35.5)	536(42.4)	576(42.8)	<0.001
	1-4/month	4985(38.7)	628(39.2)	1150(43.0)		5853(39.3)	450(41.3)	460(39.4)	
	≥2/week	3304(25.8)	293(18.4)	513(19.4)		3723(25.2)	175(16.3)	212(17.8)	
Physical activity, n(%)[3]	No	7170(49.3)	888(49.1)	1346(43.5)	<0.001	8206(48.4)	583(48.5)	615(47.0)	0.690
	Yes	6494(50.7)	813(50.9)	1495(56.5)		7591(51.6)	578(51.5)	633(53.0)	
Total energy (kcal)[4]		2071.5±8.8	2123.5±21.6	2249.8±16.3	<0.001	2090.2±8.3	2153.4±26.9	2252.7±25.4	<0.001
Percentage from energy[4]									
Carbohydrate		60.7±0.1	59.4±0.3	58.9±0.2	<0.001	60.2±0.1	62.0±0.4	60.7±0.4	<0.001
Protein		14.3±0.0	14.4±0.1	14.6±0.1	0.007	14.3±0.0	14.1±0.1	14.4±0.1	0.365
Fat		20.0±0.1	22.1±0.3	22.9±0.2	<0.001	20.6±0.1	20.5±0.3	21.4±0.3	0.010
Milk (g/day)[5]		0.2±0.1	113.7±1.9	306.2±3.5	<0.001	58.9±1.3	57.8±4.6	64.1±4.6	0.521
Low fat/skimmed milk (g/day)[5]		0.0±0.0	23.2±2.8	39.3±2.5	<0.001	7.8±0.5	9.3±1.8	14.5±2.3	0.014
Yogurt (g/day)[5]		18.8±0.7	22.5±2.3	18.8±1.4	0.276	-0.0±0.0	74.6±1.1	223.7±4.3	<0.001
Calcium (mg)[5]		465.2±2.5	568.6±6.5	759.2±6.5	<0.001	504.2±2.6	561.5±9.8	713.8±9.3	<0.001
Elderly (≥65 years)		4196	391	526		4371	435	307	
Age (years)[2]		72.2±0.1	70.9±0.3	71.1±0.2	<0.001	72.0±0.1	72.2±0.3	71.4±0.4	0.184
BMI (kg/m²)[2]		24.1±0.1	24.3±0.2	24.0±0.2	0.482	24.1±0.1	24.2±0.2	23.9±0.2	0.490
Sex, n(%)	Men	1862(46.4)	136(33.8)	202(39.5)	<0.001	1918(45.5)	168(41.6)	114(38.0)	0.029
	Women	2334(53.6)	255(66.2)	324(60.5)		2453(54.5)	267(58.4)	193(62.0)	

Education, n(%)	≤Middle school	3144(73.4)	257(62.6)	338(60.5)	<0.001	3249(72.4)	311(70.0)	179(55.7)	<0.001
	High school	693(17.4)	77(21.8)	113(23.4)		731(17.7)	77(19.1)	75(27.0)	
	≥College	347(9.2)	57(15.6)	74(16.1)		380(9.9)	46(10.8)	52(17.3)	
Household income, n(%)	Lowest	2007(45.5)	155(39.0)	191(34.7)	<0.001	2042(44.4)	201(45.4)	110(35.0)	0.052
	Lower middle	1135(27.3)	116(26.3)	142(24.2)		1183(26.9)	112(25.4)	98(28.8)	
	Upper middle	604(15.8)	62(19.0)	106(21.2)		659(16.6)	68(15.6)	45(16.9)	
	Highest	429(11.5)	57(15.7)	83(19.9)		464(12.1)	53(13.6)	52(19.3)	
Smoking, n(%)	Non-smoker	2568(59.7)	273(71.4)	358(66.9)	<0.001	2694(60.6)	293(63.9)	212(68.2)	0.029
	Former smoker	1212(29.9)	98(23.0)	122(24.6)		1246(29.0)	110(29.5)	76(25.0)	
	Current smoker	416(10.4)	20(5.5)	46(8.5)		431(10.4)	32(6.6)	19(6.7)	
Alcohol consumption, n(%)	Never/ rarely	2658(62.5)	254(64.7)	333(64.0)	0.081	2746(62.1)	303(69.5)	196(64.3)	0.049
	1-4/month	778(18.9)	80(21.6)	113(21.3)		832(19.5)	75(17.4)	64(20.5)	
	≥2/week	760(18.6)	57(13.7)	80(14.7)		793(18.4)	57(13.2)	47(15.2)	
Physical activity, n(%)[3]	No	2850(66.6)	253(62.1)	330(60.3)	0.019	2964(66.3)	284(62.6)	185(60.5)	0.099
	Yes	1346(33.4)	138(37.9)	196(39.7)		1407(33.7)	151(37.4)	122(39.5)	
Total energy (kcal)[4]		1716.7±13.3	1804.1±32.3	1865.0±32.4	<0.001	1730.4±12.6	1715.2±38.7	1893.0±40.6	0.001
Percentage from energy[4]									
Carbohydrate		71.3±0.2	68.9±0.7	67.3±0.5	<0.001	70.6±0.2	72.9±0.6	68.9±0.6	<0.001
Protein		12.9±0.1	13.3±0.2	13.6±0.2	<0.001	13.0±0.1	12.5±0.2	13.4±0.2	0.011
Fat		13.1±0.1	15.4±0.4	17.1±0.4	<0.001	13.5±0.1	12.8±0.4	16.4±0.5	<0.001
Milk (g/day)[5]		0.1±0.1	127.9±3.2	282.9±5.7	<0.001	39.5±1.8	38.1±4.5	32.0±6.4	0.524
Low fat/skimmed milk (g/day)[5]		0.1±0.1	28.3±5.0	63.4±8.8	<0.001	9.0±1.2	10.4±3.0	3.3±3.1	0.196
Yogurt (g/day)[5]		18.1±1.1	24.4±4.5	16.7±3.2	0.361	0.0±0.0	76.6±1.7	205.4±7.7	<0.001
Calcium (mg)[5]		405.7±4.5	557.6±13.7	691.4±15.1	<0.001	433.8±4.9	472.0±13.0	598.7±16.9	<0.001

[1] p values were obtained from the PROC SURVEYREG procedure for continuous variables and Chi-squared tests for categorical variables.

[2] Values are presented as means ± standard error (SE).

[3] Physical activity was defined as ≥150 min/week of moderate physical activity, ≥75 min/week of vigorous physical activity, or ≥150 min/week of a combination of moderate and vigorous physical activity (1 min of vigorous physical activity was considered 2 min of moderate physical activity)

[4] Values are presented as means ± standard error (SE). Adjusted for age (continuous), BMI (continuous), education (≤middle school, high school, or ≥college), household income (lowest, lower middle, upper middle, or highest), smoking (non-smoker, former smoker, or current smoker), alcohol consumption (never/rarely, 1-4/month, or ≥2/week), and physical activity (yes or no).

[5] Values are presented as means ± standard error (SE). Adjusted for age (continuous), BMI (continuous), education (≤middle school, high school, or ≥college), household income (lowest, lower middle, upper middle, or highest), smoking (non-smoker, former smoker, or current smoker), alcohol consumption (never/rarely, 1-4/month, or ≥2/week), physical activity (yes or no), and total energy (continuous).

Supplementary Table S2. Gender-specific general characteristics according to milk consumption in the Korean adult and elderly population.

		**Men**				**Women**			
		**Milk consumption (servings/day)**			***P* value[1]**	**Milk consuption (servings/day)**			***P* value[1]**
		0	0<to<1	≥1		0	0<to<1	≥1	
Adults (19-64 years)		5855	519	1114		7809	1182	1727	
Age (years)[2]		41.2±0.2	40.2±0.6	38.5±0.4	<0.001	42.1±0.2	42.1±0.4	40.6±0.4	0.001
BMI (kg/m^2)[2]		24.7±0.1	24.6±0.2	24.5±0.1	0.346	23.1±0.1	22.6±0.1	22.7±0.1	<0.001
Education, n(%)	≤Middle school	920(11.9)	43(5.4)	97(6.8)	<0.001	1668(17.9)	187(13.0)	259(12.4)	<0.001
	High school	2258(41.0)	177(35.6)	431(41.7)		3026(40.4)	410(36.0)	666(39.7)	
	≥College	2674(47.1)	299(59.0)	586(51.5)		3110(41.7)	584(51.0)	802(47.8)	
Household income, n(%)	Lowest	513(8.6)	33(5.8)	69(5.9)	0.004	780(9.5)	79(6.4)	123(7.5)	<0.001
	Lower middle	1351(22.6)	104(18.7)	247(22.5)		1978(25.0)	256(21.4)	407(22.1)	
	Upper middle	1884(32.5)	160(31.9)	364(34.1)		2430(31.4)	383(33.0)	549(32.4)	
	Highest	2097(36.4)	219(43.6)	432(37.6)		2600(34.1)	464(39.2)	644(38.0)	
Smoking, n(%)	Non-smoker	1428(26.5)	150(30.9)	330(32.0)	<0.001	6901(87.4)	1064(88.5)	1533(88.4)	0.168
	Former smoker	1992(31.4)	194(36.0)	396(33.4)		450(6.1)	73(7.0)	108(6.2)	
	Current smoker	2435(42.1)	175(33.1)	388(34.6)		458(6.5)	45(4.6)	86(5.4)	
Alcohol consumption, n(%)	Never/ rarely	1417(24.1)	140(26.7)	290(26.4)	<0.001	3958(48.2)	640(52.7)	888(48.5)	<0.001
	1-4/month	2241(40.3)	222(44.7)	489(44.9)		2744(37.0)	406(35.6)	661(41.1)	
	≥2/week	2197(35.7)	157(28.5)	335(28.7)		1107(14.8)	136(11.7)	178(10.4)	
Physical activity, n(%)[3]	No	2878(46.4)	238(43.0)	456(38.1)	<0.001	4292(52.5)	650(53.2)	890(48.6)	0.025
	Yes	2977(53.6)	281(57.0)	658(61.9)		3517(47.5)	532(46.8)	837(51.4)	
Total energy (kcal)[4]		2397.1±13.8	2431.0±41.7	2595.5±26.9	<0.001	1731.8±9.2	1793.5±22.1	1890.0±18.1	<0.001
Percentage from energy[4]									
Carbohydrate		58.4±0.2	58.6±0.6	57.2±0.4	0.020	63.1±0.2	60.8±0.4	60.8±0.3	<0.001
Protein		14.3±0.1	14.2±0.2	14.5±0.1	0.466	14.2±0.1	14.5±0.1	14.6±0.1	0.001
Fat		19.9±0.1	21.6±0.4	22.9±0.3	<0.001	20.1±0.1	22.4±0.3	22.9±0.2	<0.001
Milk (g/day)[5]		0.3±0.1	108.8±3.4	322.8±6.2	<0.001	0.2±0.1	115.8±2.0	289.9±3.5	<0.001
Low fat/skimmed milk (g/day)[5]		-0.0±0.0	20.2±4.7	33.6±3.5	<0.001	0.0±0.1	25.6±2.9	44.8±3.2	<0.001
Yogurt (g/day)[5]		18.1±1.0	20.3±3.2	17.5±2.1	0.757	19.6±0.8	24.1±2.9	19.9±1.8	0.317
Calcium (mg)[5]		518.4±3.7	608.8±11.8	821.6±10.3	<0.001	409.9±3.0	521.0±6.7	694.4±7.5	<0.001
Elderly (≥65 years)		1862	136	202		2334	255	324	
Age (years)[2]		72.0±0.1	70.7±0.5	70.8±0.4	0.001	72.3±0.1	71.1±0.3	71.3±0.3	<0.001
BMI (kg/m^2)[2]		23.7±0.1	24.3±0.3	23.5±0.2	0.034	24.5±0.1	24.3±0.2	24.4±0.2	0.673
Education, n(%)	≤Middle school	1122(59.8)	61(40.6)	95(45.0)	<0.001	2022(85.1)	196(73.8)	243(70.5)	<0.001
	High school	466(24.7)	36(29.8)	61(32.3)		227(11.0)	41(17.8)	52(17.6)	
	≥College	269(15.5)	39(29.6)	45(22.7)		78(3.8)	18(8.4)	29(11.9)	

Household income, n(%)	Lowest	771(39.1)	39(28.9)	58(29.6)	0.020	1236(51.1)	116(44.0)	133(38.0)	<0.001
	Lower middle	544(28.6)	47(31.3)	56(25.2)		591(26.1)	69(23.8)	86(23.5)	
	Upper middle	320(18.5)	23(21.8)	48(25.4)		284(13.4)	39(17.6)	58(18.5)	
	Highest	220(13.8)	26(18.0)	39(19.7)		209(9.5)	31(14.6)	44(20.0)	
Smoking, n(%)	Non-smoker	350(19.2)	30(25.0)	54(25.3)	0.162	2218(94.8)	243(95.2)	304(94.0)	0.830
	Former smoker	1143(61.0)	88(61.2)	112(57.6)		69(3.0)	10(3.6)	10(3.1)	
	Current smoker	369(19.8)	18(13.9)	36(17.2)		47(2.3)	2(1.3)	10(2.9)	
Alcohol consumption, n(%)	Never/ rarely	746(40.1)	50(38.3)	76(39.3)	0.666	1912(81.9)	204(78.2)	257(80.1)	0.426
	1-4/month	472(25.7)	42(29.7)	62(30.0)		306(13.1)	38(17.5)	51(15.5)	
	≥2/week	644(34.2)	44(32.0)	64(30.7)		116(5.0)	13(4.3)	16(4.3)	
Physical activity, n(%)[3]	No	1141(59.6)	74(49.2)	110(52.2)	0.025	1709(72.7)	179(68.7)	220(65.6)	0.053
	Yes	721(40.4)	62(50.8)	92(47.8)		625(27.3)	76(31.3)	104(34.4)	
Total energy (kcal)[4]		1966.2±20.2	2097.6±56.1	2161.2±51.9	0.001	1513.7±15.4	1577.7±36.9	1631.4±38.6	0.010
Percentage from energy[4]									
Carbohydrate		68.2±0.3	66.1±1.1	65.8±1.0	0.017	73.9±0.2	71.2±0.8	68.8±0.6	<0.001
Protein		13.3±0.1	13.7±0.3	13.4±0.2	0.573	12.6±0.1	13.0±0.2	13.7±0.2	<0.001
Fat		13.7±0.2	15.6±0.7	16.9±0.6	<0.001	12.6±0.2	15.1±0.6	17.1±0.5	<0.001
Milk (g/day)[5]		0.1±0.1	123.4±5.0	291.6±9.7	<0.001	0.0±0.2	130.2±4.2	277.2±6.6	<0.001
Low fat/skimmed milk (g/day)[5]		0.1±0.1	45.8±10.3	48.8±10.5	<0.001	0.1±0.2	19.9±4.4	73.0±12.7	<0.001
Yogurt (g/day)[5]		16.2±1.5	24.7±8.4	15.1±4.0	0.586	19.7±1.4	24.6±5.3	17.8±4.2	0.618
Calcium (mg)[5]		471.7±8.0	604.2±27.5	765.8±29.5	<0.001	352.4±4.9	513.7±15.0	632.3±14.9	<0.001

[1] p values were obtained from the PROC SURVEYREG procedure for continuous variables and Chi-squared tests for categorical variables.

[2] Values are presented as means ± standard error (SE).

[3] Physical activity was defined as ≥150 min/week of moderate physical activity, ≥75 min/week of vigorous physical activity, or ≥150 min/week of a combination of moderate and vigorous physical activity (1 min of vigorous physical activity was considered 2 min of moderate physical activity)

[4] Values are presented as means ± standard error (SE). Adjusted for age (continuous), BMI (continuous), education (≤middle school, high school, or ≥college), household income (lowest, lower middle, upper middle, or highest), smoking (non-smoker, former smoker, or current smoker), alcohol consumption (never/rarely, 1-4/month, or ≥2/week), and physical activity (yes or no).

[5] Values are presented as means ± standard error (SE). Adjusted for age (continuous), BMI (continuous), education (≤middle school, high school, or ≥college), household income (lowest, lower middle, upper middle, or highest), smoking (non-smoker, former smoker, or current smoker), alcohol consumption (never/rarely, 1-4/month, or ≥2/week), physical activity (yes or no), and total energy (continuous).

Supplementary Table S3. Gender-specific general characteristics according to yogurt consumption in the Korean adult and elderly population.

		Men				Women			
		Yogurt consumption (servings/day)			*P* value[1]	Yogurt consuption (servings/day)			*P* value[1]
		0	0<to<1	≥1		0	0<to<1	≥1	
Adults (19-64 years)		6665	353	470		9132	808	778	
Age (years)[2]		40.6±0.2	41.9±0.8	41.9±0.6	0.030	41.6±0.2	43.9±0.5	43.3±0.6	<0.001
BMI (kg/m^2)[2]		24.6±0.0	24.2±0.2	24.6±0.2	0.172	23.0±0.0	22.9±0.1	22.7±0.1	0.145
Education, n(%)	≤Middle school	971(11.1)	44(8.7)	45(6.6)	0.001	1793(16.3)	179(18.1)	142(16.0)	0.795
	High school	2569(41.0)	130(40.6)	167(36.0)		3501(39.9)	308(38.8)	293(39.2)	
	≥College	3122(47.9)	179(50.7)	258(57.4)		3832(43.7)	321(43.1)	343(44.8)	
Household income, n(%)	Lowest	574(8.3)	13(3.9)	28(6.0)	<0.001	859(9.1)	73(8.7)	50(5.8)	0.008
	Lower middle	1550(22.9)	73(19.3)	79(15.9)		2253(24.3)	198(22.9)	190(23.6)	
	Upper middle	2158(32.9)	104(30.5)	146(31.4)		2897(32.0)	257(32.2)	208(28.6)	
	Highest	2371(35.9)	161(46.2)	216(46.7)		3103(34.7)	278(36.3)	327(42.0)	
Smoking, n(%)	Non-smoker	1668(27.1)	99(29.1)	141(33.3)	0.004	8046(87.2)	740(90.1)	712(91.2)	0.026
	Former smoker	2274(31.8)	123(32.3)	185(35.4)		561(6.5)	36(5.0)	34(4.5)	
	Current smoker	2723(41.1)	131(38.6)	144(31.3)		525(6.3)	32(4.9)	32(4.3)	
Alcohol consumption, n(%)	Never/ rarely	1627(24.3)	83(23.7)	137(29.9)	<0.001	4594(47.8)	453(54.6)	439(55.2)	<0.001
	1-4/month	2584(40.7)	166(49.0)	202(43.5)		3269(37.8)	284(36.3)	258(35.6)	
	≥2/week	2454(35.0)	104(27.3)	131(26.6)		1269(14.5)	71(9.2)	81(9.3)	
Physical activity, n(%)[3]	No	3205(45.2)	158(43.8)	209(42.2)	0.484	5001(52.0)	425(51.6)	406(51.7)	0.969
	Yes	3460(54.8)	195(56.2)	261(57.8)		4131(48.0)	383(48.4)	372(48.3)	
Total energy (kcal)[4]		2417.5±13.0	2470.8±51.1	2575.9±41.6	0.001	1748.8±8.2	1813.3±26.6	1914.7±26.6	<0.001
Percentage from energy[4]									
Carbohydrate		58.0±0.2	60.6±0.7	59.5±0.6	<0.001	62.4±0.1	63.7±0.4	62.0±0.4	0.008
Protein		14.4±0.1	14.4±0.2	14.4±0.2	0.949	14.3±0.1	14.0±0.2	14.3±0.2	0.065
Fat		20.5±0.1	20.5±0.5	20.8±0.4	0.705	20.7±0.1	20.6±0.3	22.0±0.3	0.001
Milk (g/day)[5]		56.8±1.9	66.2±8.7	59.4±7.3	0.543	61.2±1.6	52.7±4.4	68.2±5.5	0.054
Low fat/skimmed milk (g/day)[5]		6.5±0.8	7.6±2.8	6.6±1.9	0.933	9.2±0.7	11.1±2.4	22.4±3.9	0.003
Yogurt (g/day)[5]		-0.0±0.0	74.0±1.9	233.7±6.8	<0.001	-0.0±0.0	74.7±1.2	214.0±5.2	<0.001
Calcium (mg)[5]		554.3±3.8	633.3±17.6	765.7±15.9	<0.001	452.1±3.0	495.0±10.6	657.7±10.5	<0.001
Elderly (≥65 years)		1918	168	114		2453	267	193	
Age (years)[2]		71.9±0.1	72.0±0.4	71.2±0.5	0.413	72.1±0.1	72.3±0.4	71.6±0.4	0.380
BMI (kg/m^2)[2]		23.7±0.1	23.7±0.2	23.9±0.3	0.714	24.5±0.1	24.6±0.2	23.9±0.3	0.101
Education, n(%)	≤Middle school	1149(58.6)	89(56.5)	40(36.3)	0.001	2100(83.9)	222(79.7)	139(67.7)	<0.001
	High school	475(25.1)	49(27.0)	39(34.9)		256(11.5)	28(13.5)	36(22.2)	
	≥College	288(16.3)	30(16.5)	35(28.9)		92(4.6)	16(6.8)	17(10.1)	

Household income, n(%)	Lowest	780(38.5)	62(36.5)	26(25.3)	0.055	1262(49.3)	139(51.6)	84(41.2)	0.169
	Lower middle	555(28.0)	55(33.2)	37(29.7)		628(26.0)	57(19.8)	61(28.2)	
	Upper middle	343(19.7)	25(14.4)	23(20.7)		316(14.1)	43(16.6)	22(14.5)	
	Highest	232(13.9)	25(15.9)	28(24.3)		232(10.7)	28(12.0)	24(16.1)	
Smoking, n(%)	Non-smoker	375(20.2)	31(17.7)	28(22.5)	0.244	2319(94.4)	262(96.8)	184(96.3)	0.577
	Former smoker	1165(59.9)	108(68.4)	70(62.1)		81(3.2)	2(1.8)	6(2.3)	
	Current smoker	378(19.9)	29(13.9)	16(15.4)		53(2.4)	3(1.4)	3(1.4)	
Alcohol consumption, n(%)	Never/ rarely	756(39.7)	71(42.4)	45(40.5)	0.668	1990(80.8)	232(88.8)	151(78.8)	0.032
	1-4/month	495(25.9)	47(28.8)	34(29.3)		337(14.1)	28(9.2)	30(15.2)	
	≥2/week	667(34.4)	50(28.8)	35(30.2)		126(5.1)	7(2.0)	12(6.0)	
Physical activity, n(%)[3]	No	1164(58.9)	102(57.1)	59(51.2)	0.375	1800(72.5)	182(66.5)	126(66.2)	0.070
	Yes	754(41.1)	66(42.9)	55(48.8)		653(27.5)	85(33.5)	67(33.8)	
Total energy (kcal)[4]		1989.2±19.0	1949.4±69.7	2091.6±63.0	0.233	1519.5±14.8	1520.7±40.9	1724.8±49.9	<0.001
Percentage from energy[4]									
Carbohydrate		67.6±0.3	71.1±1.0	66.9±1.0	0.003	73.1±0.2	74.7±0.7	70.7±0.8	0.001
Protein		13.4±0.1	12.9±0.3	14.0±0.3	0.057	12.8±0.1	12.3±0.2	12.9±0.2	0.095
Fat		14.0±0.2	13.5±0.7	16.4±0.7	0.002	13.2±0.2	12.3±0.5	16.2±0.6	<0.001
Milk (g/day)[5]		33.9±2.5	33.8±6.8	26.7±8.2	0.703	44.1±2.5	41.5±5.8	36.1±8.2	0.628
Low fat/skimmed milk (g/day)[5]		7.0±1.3	11.0±5.4	0.9±2.9	0.127	10.7±1.9	9.7±3.4	4.6±4.6	0.493
Yogurt (g/day)[5]		-0.0±0.1	74.2±2.3	213.3±14.3	<0.001	0.1±0.1	78.3±2.2	200.3±7.8	<0.001
Calcium (mg)[5]		495.8±8.5	524.6±23.3	648.2±30.5	<0.001	383.7±5.4	429.7±14.5	555.11±18.3	<0.001

[1] p values were obtained from the PROC SURVEYREG procedure for continuous variables and Chi-squared tests for categorical variables.

[2] Values are presented as means ± standard error (SE).

[3] Physical activity was defined as ≥150 min/week of moderate physical activity, ≥75 min/week of vigorous physical activity, or ≥150 min/week of a combination of moderate and vigorous physical activity (1 min of vigorous physical activity was considered 2 min of moderate physical activity)

[4] Values are presented as means ± standard error (SE). Adjusted for age (continuous), BMI (continuous), education (≤middle school, high school, or ≥college), household income (lowest, lower middle, upper middle, or highest), smoking (non-smoker, former smoker, or current smoker), alcohol consumption (never/rarely, 1-4/month, or ≥2/week), and physical activity (yes or no).

[5] Values are presented as means ± standard error (SE). Adjusted for age (continuous), BMI (continuous), education (≤middle school, high school, or ≥college), household income (lowest, lower middle, upper middle, or highest), smoking (non-smoker, former smoker, or current smoker), alcohol consumption (never/rarely, 1-4/month, or ≥2/week), physical activity (yes or no), and total energy (continuous).

Supplementary Table S4. Multivariable-adjusted odds ratio (ORs) and 95% confidence intervals (CIs) for metabolic syndrome components according to milk consumption in Korean adult and elderly population.

	Milk consumption (servings/day)			*P* trend[1]
	0	0< to <1	≥1	
All adults				
Abdominal obesity	1.0(reference)	0.94(0.74-1.19)	0.97(0.81-1.16)	0.663
Elevated blood pressure	1.0(reference)	1.04(0.90-1.21)	0.97(0.86-1.10)	0.786
Low HDL-cholesterol	1.0(reference)	0.98(0.86-1.11)	0.97(0.87-1.08)	0.566
Hypertriglyceridemia	1.0(reference)	1.02(0.88-1.18)	0.84(0.75-0.95)	0.009
Hyperglycemia	1.0(reference)	1.12(0.97-1.30)	0.96(0.85-1.07)	0.770
Men				
Abdominal obesity	1.0(reference)	1.02(0.72-1.45)	1.00(0.78-1.27)	0.994
Elevated blood pressure	1.0(reference)	1.14(0.91-1.43)	1.03(0.87-1.21)	0.598
Low HDL-cholesterol	1.0(reference)	0.97(0.77-1.21)	1.05(0.88-1.25)	0.677
Hypertriglyceridemia	1.0(reference)	1.00(0.81-1.24)	0.77(0.66-0.90)	0.002
Hyperglycemia	1.0(reference)	1.20(0.95-1.51)	0.97(0.82-1.14)	0.998
Women				
Abdominal obesity	1.0(reference)	0.83(0.62-1.11)	0.92(0.72-1.19)	0.359
Elevated blood pressure	1.0(reference)	0.89(0.73-1.08)	0.87(0.73-1.03)	0.069
Low HDL-cholesterol	1.0(reference)	0.99(0.85-1.16)	0.92(0.81-1.05)	0.270
Hypertriglyceridemia	1.0(reference)	1.03(0.85-1.24)	0.96(0.81-1.14)	0.749
Hyperglycemia	1.0(reference)	1.08(0.91-1.30)	0.95(0.81-1.11)	0.747
All elderly people				
Abdominal obesity	1.0(reference)	0.72(0.50-1.04)	0.86(0.64-1.15)	0.101
Elevated blood pressure	1.0(reference)	1.11(0.86-1.45)	1.02(0.80-1.30)	0.649
Low HDL-cholesterol	1.0(reference)	0.85(0.66-1.09)	0.69(0.54-0.88)	0.002
Hypertriglyceridemia	1.0(reference)	0.90(0.68-1.19)	0.96(0.74-1.23)	0.527
Hyperglycemia	1.0(reference)	1.09(0.85-1.41)	0.96(0.77-1.20)	0.987
Men				
Abdominal obesity	1.0(reference)	0.71(0.38-1.33)	0.77(0.46-1.27)	0.191
Elevated blood pressure	1.0(reference)	1.15(0.75-1.76)	1.10(0.76-1.60)	0.507
Low HDL-cholesterol	1.0(reference)	0.77(0.49-1.21)	0.85(0.57-1.28)	0.299
Hypertriglyceridemia	1.0(reference)	0.87(0.56-1.35)	1.07(0.73-1.55)	0.907
Hyperglycemia	1.0(reference)	0.91(0.60-1.39)	0.89(0.62-1.28)	0.482
Women				
Abdominal obesity	1.0(reference)	0.73(0.48-1.11)	0.95(0.66-1.37)	0.362
Elevated blood pressure	1.0(reference)	1.09(0.78-1.51)	0.98(0.71-1.35)	0.898
Low HDL-cholesterol	1.0(reference)	0.86(0.64-1.17)	0.63(0.48-0.84)	0.002
Hypertriglyceridemia	1.0(reference)	0.89(0.64-1.25)	0.91(0.66-1.25)	0.413
Hyperglycemia	1.0(reference)	1.21(0.87-1.68)	1.02(0.78-1.35)	0.485

[1] *P* value were obtained from a regression model using the PROC SURVEYLOGISTIC procedure.

[2] Adjusted for age (continuous), sex, BMI (continuous), education (≤middle school, high school, or ≥college), household income (lowest, lower middle, upper middle, or highest), smoking (non-smoker, former smoker, or current smoker), alcohol consumption (never/rarely, 1-4/month, or ≥2/week), physical activity (yes or no), and total energy (continuous).

Supplementary Table S5. Multivariable-adjusted odds ratio (ORs) and 95% confidence intervals (CIs) for metabolic syndrome components according to yogurt consumption in Korean adult and elderly population.

	**Yogurt consumption (servings/day)**			***P* trend**[1]
	**0**	**0< to <1**	**≥1**	
All adults				
Abdominal obesity	1.0(reference)	0.98(0.74-1.29)	0.94(0.74-1.20)	0.598
Elevated blood pressure	1.0(reference)	0.94(0.78-1.11)	0.98(0.83-1.16)	0.720
Low HDL-cholesterol	1.0(reference)	1.08(0.92-1.28)	0.95(0.81-1.11)	0.671
Hypertriglyceridemia	1.0(reference)	0.94(0.78-1.13)	0.89(0.76-1.05)	0.139
Hyperglycemia	1.0(reference)	0.84(0.70-1.01)	0.82(0.70-0.96)	0.007
Men				
Abdominal obesity	1.0(reference)	0.96(0.62-1.47)	0.96(0.68-1.35)	0.781
Elevated blood pressure	1.0(reference)	0.87(0.66-1.14)	0.91(0.72-1.17)	0.383
Low HDL-cholesterol	1.0(reference)	1.17(0.86-1.57)	1.00(0.77-1.29)	0.865
Hypertriglyceridemia	1.0(reference)	0.91(0.70-1.19)	0.87(0.69-1.09)	0.185
Hyperglycemia	1.0(reference)	0.83(0.62-1.12)	0.88(0.69-1.11)	0.209
Women				
Abdominal obesity	1.0(reference)	0.96(0.69-1.33)	0.86(0.64-1.17)	0.331
Elevated blood pressure	1.0(reference)	0.95(0.77-1.18)	1.07(0.86-1.33)	0.650
Low HDL-cholesterol	1.0(reference)	1.03(0.87-1.24)	0.91(0.75-1.09)	0.364
Hypertriglyceridemia	1.0(reference)	0.94(0.73-1.21)	0.92(0.72-1.18)	0.455
Hyperglycemia	1.0(reference)	0.85(0.69-1.06)	0.76(0.61-0.94)	0.006
All elderly people				
Abdominal obesity	1.0(reference)	0.97(0.68-1.40)	0.96(0.66-1.40)	0.808
Elevated blood pressure	1.0(reference)	0.89(0.69-1.14)	0.90(0.66-1.21)	0.375
Low HDL-cholesterol	1.0(reference)	0.89(0.68-1.17)	1.08(0.82-1.42)	0.778
Hypertriglyceridemia	1.0(reference)	0.78(0.61-1.01)	0.94(0.69-1.28)	0.415
Hyperglycemia	1.0(reference)	0.86(0.67-1.09)	0.77(0.58-1.02)	0.041
Men				
Abdominal obesity	1.0(reference)	1.28(0.73-2.25)	1.02(0.56-1.84)	0.750
Elevated blood pressure	1.0(reference)	0.73(0.50-1.06)	0.90(0.55-1.47)	0.413
Low HDL-cholesterol	1.0(reference)	1.01(0.66-1.56)	0.84(0.50-1.42)	0.548
Hypertriglyceridemia	1.0(reference)	0.67(0.45-1.01)	1.19(0.73-1.95)	0.832
Hyperglycemia	1.0(reference)	0.97(0.65-1.44)	0.65(0.43-0.98)	0.049
Women				
Abdominal obesity	1.0(reference)	0.86(0.56-1.32)	0.91(0.57-1.47)	0.591
Elevated blood pressure	1.0(reference)	1.05(0.76-1.47)	0.91(0.62-1.33)	0.676
Low HDL-cholesterol	1.0(reference)	0.84(0.61-1.15)	1.19(0.84-1.68)	0.516
Hypertriglyceridemia	1.0(reference)	0.85(0.62-1.17)	0.79(0.53-1.18)	0.186
Hyperglycemia	1.0(reference)	0.78(0.58-1.05)	0.86(0.60-1.23)	0.234

[1] *P* value were obtained from a regression model using the PROC SURVEYLOGISTIC procedure.

[2] Adjusted for age (continuous), sex, BMI (continuous), education (≤middle school, high school, or ≥college), household income (lowest, lower middle, upper middle, or highest), smoking (non-smoker, former smoker, or current smoker), alcohol consumption (never/rarely, 1-4/month, or ≥2/week), physical activity (yes or no), and total energy (continuous).

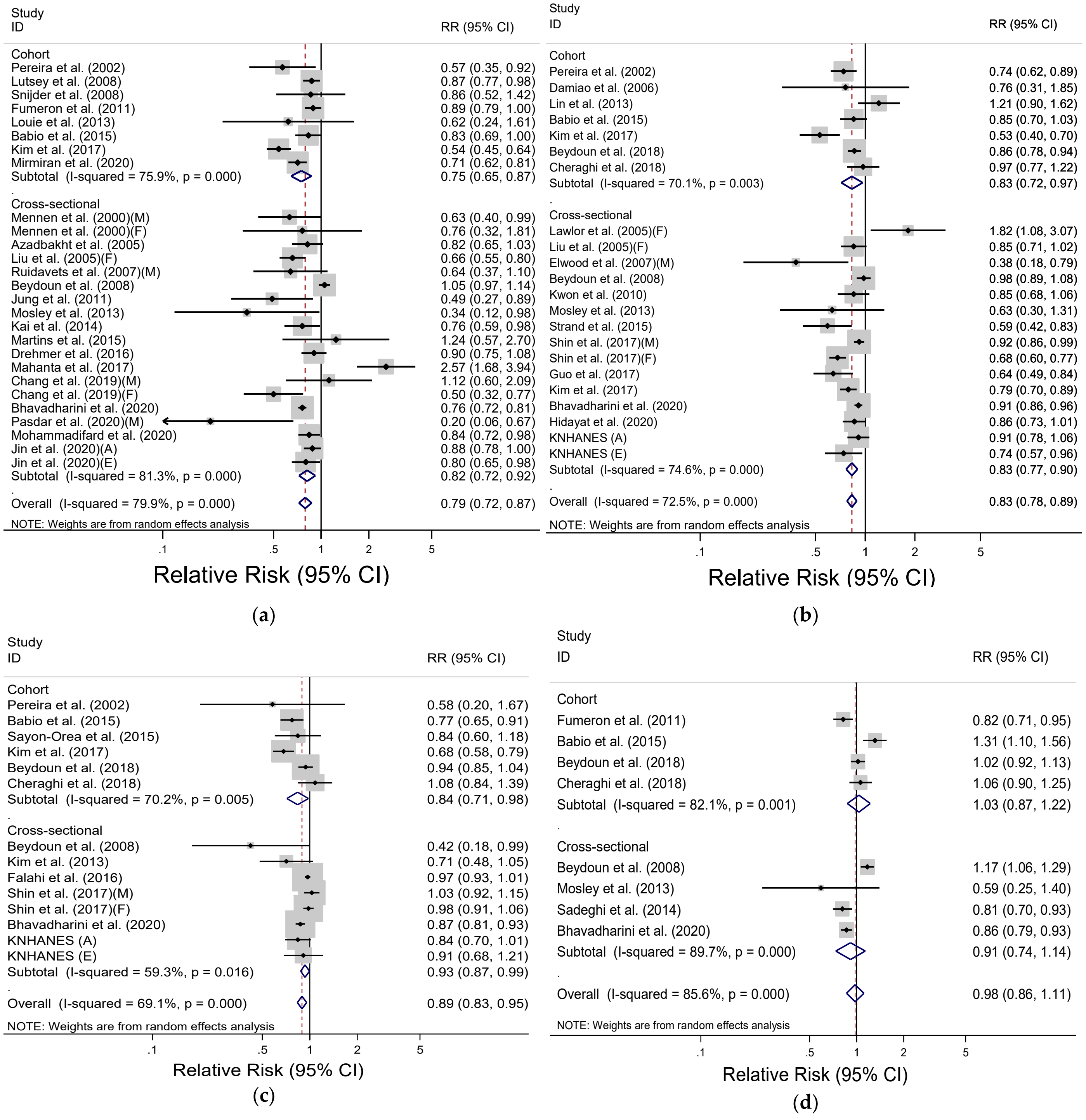

Supplementary Figure S1. Forest plot of the observational studies for risk of metabolic syndrome. RRs for the highest versus lowest consumption of (**a**) total dairy, (**b**) milk, (**c**) yogurt, and (**d**) cheese. The sizes of the squares correspond to the inverse of the variance of the natural logarithm of the RR from individual study, and the diamond indicates the pooled RR. F, female; M, male; A, adult; E, elderly
